# Unlocking the Potential of MXene in Catalysis: Decorated Mo_2_CT_*x*_ Catalyst for Ammonia Synthesis under Mild Conditions

**DOI:** 10.1021/jacs.4c03875

**Published:** 2024-07-12

**Authors:** Amanda Sfeir, Christopher E. Shuck, Alexandre Fadel, Maya Marinova, Hervé Vezin, Jean-Philippe Dacquin, Yury Gogotsi, Sébastien Royer, Said Laassiri

**Affiliations:** † CNRS, ENSCL, Centrale Lille, Univ. Artois, UMR 8181-UCCS-Unité de Catalyse et de Chimie du Solide, 27023 Université de Lille, F-59000 Lille, France; ‡ A.J. Drexel Nanomaterials Institute and Department of Materials Science and Engineering, Drexel University, Philadelphia, Pennsylvania 19104, United States; § CNRS, INRA, Centrale Lille, Université Artois, FR 2638—IMEC—Institut Michel-Eugène Chevreul, 27023 Université de Lille, 59000 Lille, France; ∥ Laboratoire de Spectroscopie pour Les Interactions La Réactivité et L’Environnement, UMR CNRS 8516-LASIRE, 27023 Université de Lille, 59000 Lille, France; ⊥ Chemical & Biochemical Sciences, Green Process Engineering (CBS), 479571 Mohammed VI Polytechnic University, UM6P, 43150 Benguerir, Morocco

## Abstract

Ammonia, which is one of the most important chemicals for the synthesis of dyes, pharmaceuticals, and fertilizers, is produced by the reaction of molecular hydrogen with nitrogen, over an iron-based catalyst at 400–500 °C under pressure of over 100 bar. Decreasing the operating temperature and pressure of this highly energy-intensive process, developed by Haber and Bosch over 100 years ago, would decrease energy consumption in the world. In this work, we used two-dimensional Mo_2_CT_*x*_ MXene as a support for a cobalt-based catalyst. The MXene functionalized by Co showed catalytic activity for ammonia synthesis from H_2_ and N_2_ at temperatures as low as 250 °C, without any pretreatment. The developed catalyst was highly active for ammonia synthesis, demonstrating a high rate of up to 9500 μmol g^–1^_active phase_ h^–1^ at 400 °C under ambient pressure in steady-state conditions, and did not suffer from any deactivation after 15 days of reaction. The apparent activation energy (*E*_a_) was found to be in the range of 68–74 kJ mol^–1^, which is in line with values reported for highly active catalysts. This improved catalyst may decrease the energy consumption in the synthesis of ammonia and its derivatives, as well as facilitate the use of ammonia as a hydrogen carrier for renewable energy storage.

## Graphical abstract

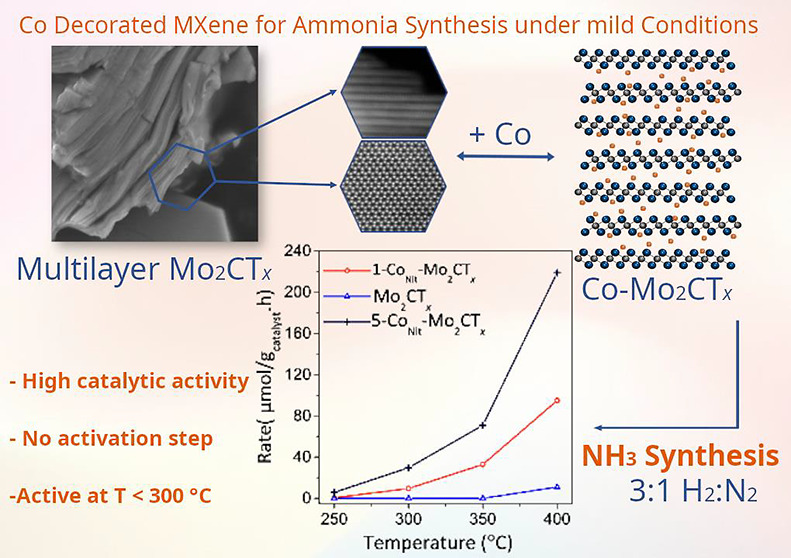

## Introduction

Ammonia is the primary nitrogen building block for most commercial and industrial nitrogen-containing molecules, including dyes, pharmaceuticals, fertilizers, etc.^1^ Additionally, ammonia can be used as a hydrogen carrier for medium- and long-term renewable energy storage.^2^ Currently, large-scale ammonia production is achieved through the Haber Bosch (H–B) process by the combination of hydrogen (H_2_), typically derived from fossil fuels, and molecular nitrogen (N_2_), over a promoted iron-based catalyst. To achieve acceptable ammonia production rates, the H–B process is operated at temperatures ranging between 400 and 500 °C at high pressure (>150 bar). While the H–B process is credited for providing a simple and cost-effective route for nitrogen-based fertilizer production, it is highly energy-intensive, accounting for ∼1% of the world’s energy production, and it relies on fossil-based resources, resulting in a staggering emission of 1–2% of global CO_2_ annually.^1,3^ There is a need for decarbonizing ammonia synthesis to minimize the carbon footprint of agriculture and contribute to global efforts to combat climate change. To this end, significant research is focused on replacing hydrogen from nonrenewable resources with green H_2_ generated from water electrolysis powered by renewable resources. However, the integration of renewable energy into the H–B process results in new challenges related to (i) intermittency of most renewable energy resources, (ii) downsizing of the H–B process to align with the scale of renewable energy sources, and (iii) high cost of green hydrogen. To alleviate these challenges, research is currently directed toward lowering operational and capital costs of green ammonia production through the use of more efficient catalysts capable of operating at lower pressures (50 bar) and temperatures (300 °C), while being capable of coping with intermittent operation conditions.

Fundamentally, producing ammonia at lower pressures is not thermodynamically prohibited, providing that the reaction takes place at temperatures below 300 °C.^4^ However, due to the high energy barrier for the activation/dissociation of the strong triple covalent N≡N bond (945 kJ mol^–1^), most developed catalysts, including the industrial H–B catalyst, operate at temperatures over 400 °C. Consequently, high-pressure operation is required to increase ammonia yields to acceptable industrial production (15% conversion per cycle). The performance of ammonia synthesis catalysts and the reaction mechanisms are dictated by the complex interaction of the catalyst surface with reactants (N_2_ and H_2_), intermediates species (NH_*x*_), and the reaction product (NH_3_).^5–8^ As such, modulating the electronic states of the active sites either by (i) doping using electron donors, such as alkaline or alkaline earth materials, (ii) alloying with transition metals, and/or (iii) controlling support-active site interaction to improve the catalytic activity and stability at lower temperatures. Co-based catalysts serve as excellent examples of how fine-tuning the properties of the active sites can lead to improvement in ammonia synthesis performance. Undoped and unsupported Co does not exhibit significant catalytic activity in ammonia synthesis due to the low adsorption energy of N_2_ on cobalt.^5^ Combining Co with transition metals (i.e., Mo, Fe, Re) modulates the catalytic performance in ammonia synthesis.^9–11^ The association of Co with Mo in Co_3_Mo_3_N resulted in a substantial increase in performance compared to the individual components [synthesis rate of 330–652 μmol h^–1^ g^–1^ at 400 °C and atmospheric pressure and weight hourly space velocity (WHSV) of 9000 mL g^–1^ h ^–1^].^12,13^ The presence of both, Co and Mo, in the (111) termination plane leads to an improved nitrogen binding energy (BE).^5^ New insights from density functional theory (DFT) calculations and ^15^N/^14^N isotopic exchange studies have highlighted the role of nitride lattice nitrogen in ammonia synthesis via routes akin to the Mars-van Krevelen (MvK) mechanism.^14–16^ The effect of supports, such as CeO_2_, BaTiO_3–*x*_H_*x*_, BaCeO_3–*x*_N_*y*_H_*z*_, and BaCa­(NH_2_)_2_, LaN,^17–20^ and/or catalytic activity promoters, such as BaO, LiH, and BaH_2_,^21–23^ in promoting the catalytic activity of Co for ammonia synthesis has also been studied and demonstrated the importance of modulating cobalt electronic states through electron transfer from dopants and/or support for achieving high catalytic activity at low temperatures. While supporting cobalt on electrides, hydrides, amides, nitrides, and oxynitrides holds great promise for developing alternative industrial catalysts to the H–B process, there are challenges related to (i) scalability of synthesis methods and (ii) reactivity under ambient conditions that must be addressed before successful implementation.

In this context, MXenes, a large 2D family of transition metal carbides, nitrides, and carbonitrides, have attracted interest as promising supports due to their superior electrical conductivity, tunable work function, high specific surface area, mechanical stability, hydrophilicity, and ability to accommodate various ions/molecules on and between their layers.^22,23^ The chemical structure of 2D MXene is M_*n*+1_X_*n*_T_*x*_ (*n* = 1, 2, 3, 4), where *M* represents an early transition metal (Mo, V, Ti, W, Nb, etc.), X is carbon and/or nitrogen and *T*_*x*_ represents the surface-terminating functional groups (−O, −OH, −F).^24,25^ Tailoring the chemical composition of MXenes by the appropriate selection of transition metal and/or surface terminations permits a precise adjustment of the density of states^25^ and work function,^26^ making MXenes attractive for catalytic applications. Furthermore, the presence of functional groups on the negatively charged surface of MXenes facilitates the adsorption of metal precursors via electrostatic interactions, making them ideal anchors for catalysts.^27–29^ With this tunable surface chemistry, as well as scalable synthesis,^30^ MXenes have the potential for use as catalysts or supports for various catalytic reactions, but the use of MXene in heterogeneous catalysis is still limited to only a few examples in literature.^31–36^ Compared with state-of-the-art materials such as electrides and lithium hydride-metal catalysts, the synthesis of MXenes is scalable.^37^ Additionally, the MXenes are easily shaped, with strategies adapted to the applications. Therefore, MXenes can be impregnated onto supports, 3D printed, spray-coated, spin-coated, or pelletized.^38^ Every step of the MXene synthesis process can then be scaled up and shaped using conventional chemical engineering principles. While more work must be done to optimize the catalyst for industrial application, our research has demonstrated the potential of Co-decorated Mo-MXenes.

In this work, we report on the performance of a Mo_2_CT_*x*_ MXene modified by cobalt in catalytic ammonia synthesis.^39^ The functionalization of MXene by Co resulted in catalytic activity in ammonia synthesis at temperatures as low as 250 °C. Co-decorated MXene was active without any pretreatment, achieving a rate of 9500 μmol g^–1^_active phase_ h^–1^ under steady-state conditions at 400 °C and without visible deactivation after 200 h in reaction.

## Results and Discussion

### Synthesis and Characterization of Mo_2_CT_*x*_ MXene Support

Multilayered Co–Mo_2_CT_*x*_ catalysts were prepared by postmodification of MXene (Mo_2_CT_*x*_). First, the parent precursor phase (Mo_2_Ga_2_C) was prepared by a reaction of Mo_2_C powder and metallic gallium (15 days at 850 °C). The formation of the Mo_2_Ga_2_C phase was confirmed by X-ray diffraction (XRD) (Figure 1a) with traces of Mo_2_C visible in the pattern background. However, this minor impurity does not affect the etching process as seen previously in the literature.^39^ Upon Ga removal by etching, the XRD showed that the (0002) peak shifted to lower 2θ from ∼9.8° to 8.1° corresponding to the modification of the interlayer spacing and formation of MXene.^40^ However, a remnant of the (0002) peak of Mo_2_Ga_2_C at 9.8° was still visible in the multilayer diffractogram, indicating the presence of a fraction of the unetched precursor phase alongside the MXene. However, the direct quantification of the MAX/MXene phases using XRD is quite challenging since the intensity of MXene diffraction peaks depends highly on the amount of water in the interlayer due to its aligning effect.^41^ In our case, the vast majority of the water is removed during the drying step, which decreases the intensity of the diffraction peaks related to the MXene phase. Complementary analysis was performed using an inductively coupled plasma optical emission spectrometer (ICP-OES) to quantify the remaining MAX phase, which was found to be 4 ± 0.5 wt % of Mo_2_Ga_2_C. This residual Ga content is common for nondelaminated multilayer MXenes.^39,42^ The typical accordion-like morphology was confirmed by SEM, suggesting the successful exfoliation of individual MXene sheets (Figure 1b). However, despite this sheet-like structure, N_2_ physisorption at 77K did not allow to measure accessible surface area (Brunauer–Emmett–Teller, BET) which falls below 1 m^2^ g^–1^. A high-angle annular dark-field scanning transmission electron microscopy (HAADF-STEM) image showing the (0002) section of Mo_2_CT_*x*_ is presented in Figure 1c, where the bright dots correspond to heavier Mo atoms compared to C atoms.

**Figure 1:**
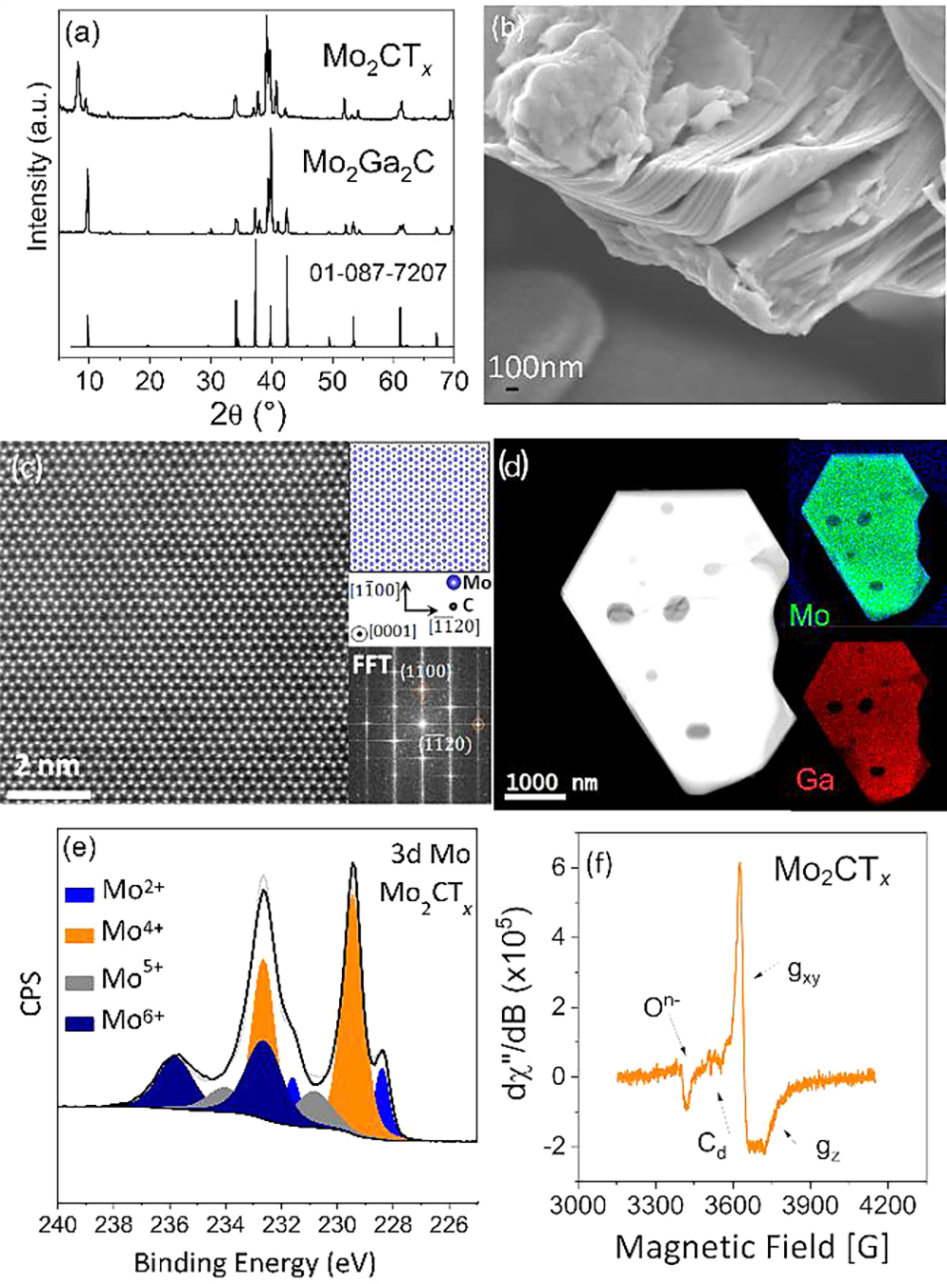
Structural and textural characterizations of Mo_2_CT_*x*_ support. (a) XRD pattern of the precursor phase (Mo_2_Ga_2_C), the MXene (Mo_2_CT_*x*_) and the reference pattern of Mo_2_Ga_2_C (01-078-7207), (b) typical SEM image of Mo_2_CT_*x*_ displaying characteristic stacked layers with 2D morphology, (c) high-resolution HAADF image and the corresponding atomic projection of the crystal lattice and Fast Fourier Transforms (FFT) for the [0001] zone axis, (d) characteristic HAADF image and the corresponding STEM-EDS intensity maps for Mo-L_a_ edge at 2.292 keV (green), C–K at 0.277 keV (blue) and Ga–K_α_ at 9.242 keV (red)—image selected for the presence of residual Ga, (e) XPS spectra in the Mo 3d region of Mo_2_CT_*x*_, and (f) EPR spectra recorded before ammonia synthesis reaction.

The chemical composition was investigated by means of energy dispersive spectroscopy (EDS) elemental mapping showing a homogeneous distribution of Mo atoms, along with some residual Ga from the parent material (Figure 1d).

Proper etching was confirmed in the high-resolution XPS spectrum of Ga 2p that shows a very low intensity of Ga peaks with less than 2.7 at. % with respect to the parent material (Figure S1), a value in agreement with residual Ga content reported in the literature and from ICP measurements on the multilayer MXene.^39,42^ Thermogravimetric analysis (TGA) was also conducted, under nitrogen, on Mo_2_CT_*x*_ multilayer (Figure S2) in order to investigate its thermal stability for high-temperature reactions. Only 0.7 wt % weight loss, correlated to water desorption and loss of some surface terminations,^43^ was reported reaching 450 °C, confirming that multilayer Mo_2_CT_*x*_ is thermally stable for reaction at 400 °C.

The identity of Mo species in Mo_2_CT_*x*_ was investigated in XPS (Figure 1e, Table S1). The spectral deconvolution of the Mo 3d shows predominately Mo^4+^, Mo^5+^, and Mo^6+^, which are attributed to the various C–Mo-T_*x*_ species (presence of −O–, −OH, and −F terminations). Their corresponding BEs are as follows: Mo^4+^ (229.45 and 232.65 eV); Mo^5+^ (230.82 and 234.02 eV); and Mo^6+^ (232.62 and 235.82 eV). The most abundant C–Mo–T_*x*_ contribution is related to the presence of Mo^4+^ (50.0 at. %), which can be associated with Mo_2_CO, Mo_2_C­(OH)_2_, Mo_2_C­(OH)­F, whereas Mo^5+^ species (Mo_2_C­(OH)­O, Mo_2_C­(F)­O) accounted for ∼12.6 at. % and Mo^6+^ (Mo_2_C­(OH)­(F)­(O), Mo_2_C­(O)_2_) for ∼27.5 at. %. Additionally, some Mo^2+^ species (Mo 3d_5/2_ at 228.4 eV) can also be observed (9.9 at. %), attributed to Mo_2_C in the carbide state and/or residual unetched Mo_2_Ga_2_C in the sample.^42^

The presence of anionic oxo, carbon defects, and Mo^5+^ species in Mo_2_CT_*x*_ MXene material was investigated using electron paramagnetic resonance (EPR) (Figure 1f). The first signal, a strong anisotropic signal at *g*_*xy*_ = 1.93 and *g*_*z*_ = 1.88, was attributed to Mo^5+^ species located in axial symmetry. The presence of Mo^5+^ is primarily associated with the existence of C–Mo-T_*x*_, a conclusion supported by XPS (Figure 1b, Table S1). A second signal, weaker in intensity, at a *g* factor of 2.06 was associated with anionic oxo species, whereas a third signal with a *g* factor value of approximately 2.0002 was attributed to carbon defects.^42,44^

### Characterization of Co–Mo_2_CT_*x*_ Catalysts

X–Co-Mo_2_CT_*x*_ catalysts were prepared by postsynthesis modification of Mo_2_CT_*x*_ by Co^2+^ at different loadings (1 and 5 wt %, using Co­(II) nitrate precursor). The integrity of the MXene structure upon cobalt impregnation was confirmed using SEM, TEM, and XRD (Figures 2, 3, and 4a). In addition, ICP measurement confirmed the Co loadings at the desired loadings ±5% (in 1–Co-Mo_2_CT_*x*_, loading of 1.05 wt % are measured for both samples, Nit or Chl, while 1.00 wt % was expected).

**Figure 2:**
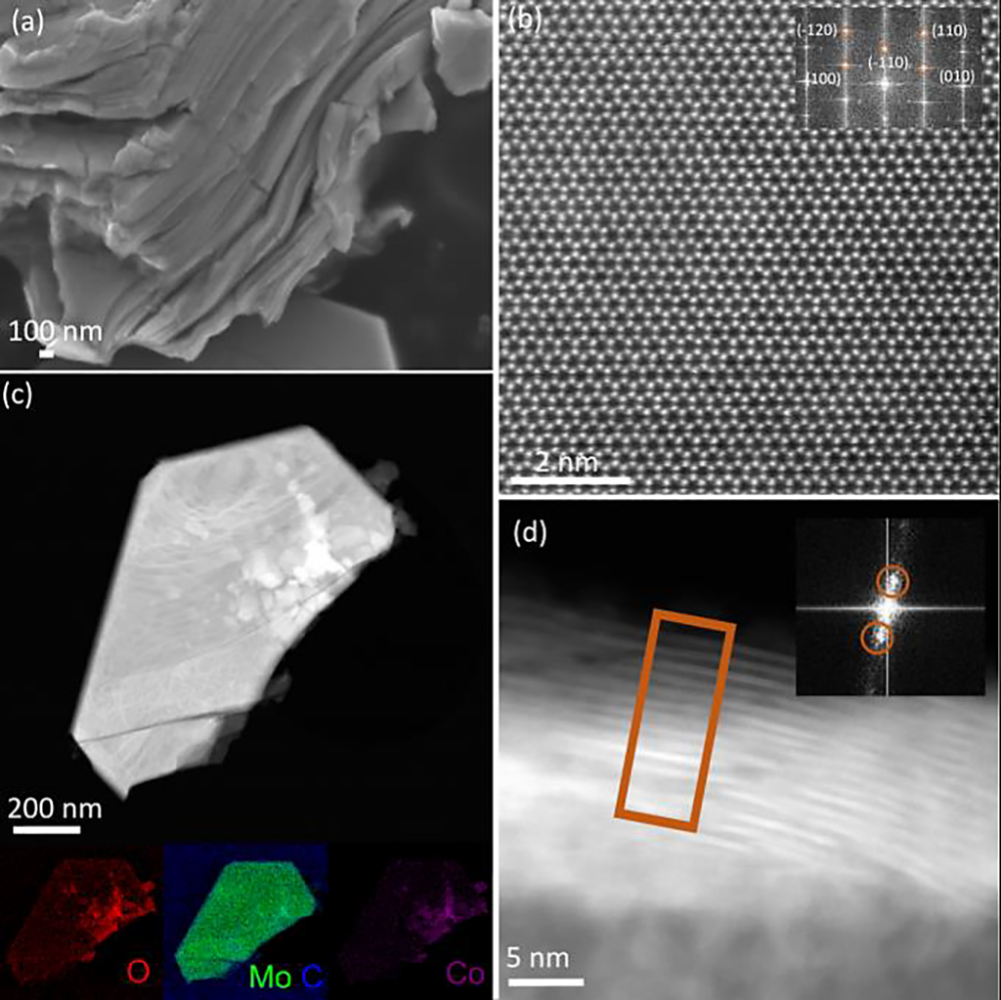
Microscopy characterization of 1-Co_Nit_-Mo_2_CT_*x*_ catalyst. (a) Typical SEM image of 1-Co_Nit_-Mo_2_CT_*x*_ displaying characteristic stacked layers with platelet morphology, (b) high-resolution HAADF-STEM image viewed along the [0001] axis, inset shows FFT pattern, and (c) characteristic HAADF image and the corresponding STEM-EDS intensity maps for Mo-L_a_ edge at 2.292 keV (green), C–K at 0.277 keV (blue), Co–K_a_ at 6.926 keV (purple), O–K at 0.525 keV (red) of 1-Co_Nit_-Mo_2_CT_*x*_, (d) HAADF-STEM image showing 1-Co_Nit_-Mo_2_CT_*x*_ MXene layers in a zone axis close to [1̅21̅0] where (0002) reflections are visible.

**Figure 3:**
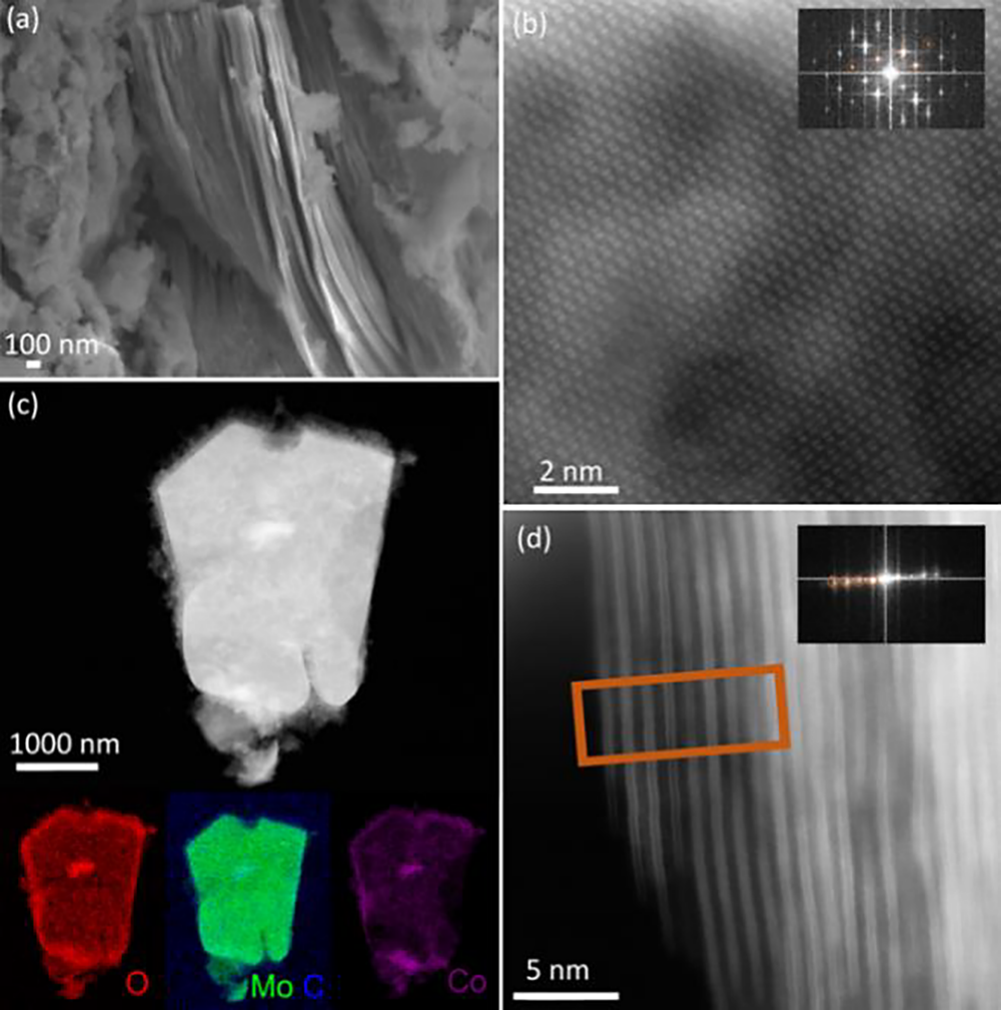
Microscopy characterization of 5-Co_Nit_-Mo_2_CT_*x*_ catalyst. (a) SEM image of 5-Co_Nit_-Mo_2_CT_*x*_ displaying characteristic stacked layers with platelet morphology covered by external cobalt phase, (b) high-resolution HAADF-STEM image and corresponding FFT in the [5̅105̅3] zone axis. (c) HAADF images and the corresponding STEM-EDS intensity maps for Mo-L_a_ edge at 2.292 keV (green), C–K at 0.277 keV (blue), Co–K_a_ at 6.926 keV (purple), O–K at 0.525 keV (red) of 5-Co_Nit_-Mo_2_CT_*x*_, (d) HAADF-STEM image showing 5-Co_Nit_-Mo_2_CT_*x*_ MXene layers in a zone axis close to [1̅21̅0] where the (0002) reflections are visible.

**Figure 4:**
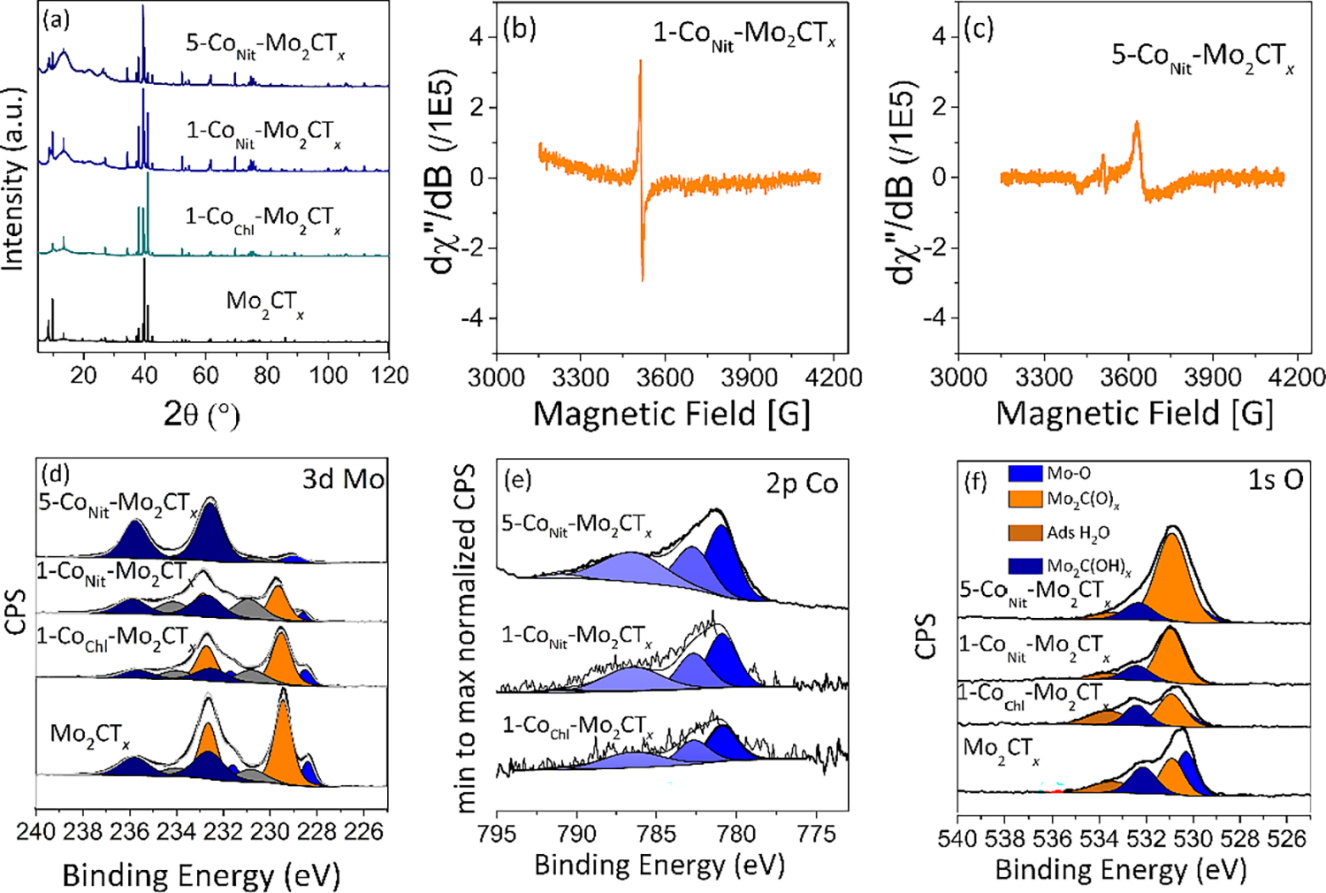
XRD and spectroscopic characterization of 1-Co_Nit_-Mo_2_CT_*x*_ and 5-Co_Nit_-Mo_2_CT_*x*_ catalysts. (a) XRD patterns of 1-Co_Nit_-Mo_2_CT_*x*_ and 5-Co_Nit_-Mo_2_CT_*x*_ catalysts, (b) EPR spectra of 1-Co_Nit_-Mo_2_CT_*x*_ recorded prior to ammonia synthesis reaction, (c) EPR spectra of 5-Co_Nit_-Mo_2_CT_*x*_ recorded prior to ammonia synthesis reaction, (d) XPS spectra in the Mo 3d region of Mo_2_CT_*x*_ of 1-Co_Nit_-Mo_2_CT_*x*_ and 5-Co_Nit_-Mo_2_CT_*x*_ catalysts, (e) XPS spectra in the Co 2p region of Mo_2_CT_*x*_ of 1-Co_Nit_-Mo_2_CT_*x*_ and 5-Co_Nit_-Mo_2_CT_*x*_ catalysts, and (f) XPS spectra in the O 1s region of Mo_2_CT_*x*_ of 1-Co_Nit_-Mo_2_CT_*x*_ and 5-Co_Nit_-Mo_2_CT_*x*_ catalysts.

In Figure 2, the accordion-like structure of MXene multilayers is retained for 1-Co_Nit_-Mo_2_CT_*x*_ with traces of the unetched precursor. STEM-EDS demonstrates good dispersion of Co species within Mo_2_CT_*x*_ (Figure 2c). At high cobalt loading, 5-Co_Nit_-Mo_2_CT_*x*_ showed an amorphous layer of cobalt oxides enveloping Mo_2_CT_*x*_ (Figure 3a). However, the typical accordion morphology distinctive of MXene multilayers was maintained at high cobalt loading. Again, and whatever the Co loading, samples did not demonstrate any measurable textural properties as evaluated by N_2_ physisorption at 77K (BET surface area <1 m^2^ g^–1^). XRD analysis was performed to further study the phase transformation upon cobalt impregnation (Figure 4a). In all samples, (0002) diffraction peaks corresponding to the MXene Mo_2_CT_*x*_ (2θ ∼ 8.21°; *d* ∼ 10.76 Å) and the unetched Mo_2_Ga_2_C (2θ ∼ 9.79°; *d* ∼ 9.03 Å) are present. However, due to the changes in plane orientation of 2D materials (*z* = 0), the intensity of the (0002) peaks decreased significantly after cobalt incorporation. At low 2θ, the broadening phenomenon observed stems from the differences in the registry of the MXene flakes as Co^2+^ is intercalated. This variation arises from the presence of water and Co^2+^ in some cases, Co^2+^ or water separately in some others, leading to nonuniform stacking and subsequent line broadening. This phenomenon has been documented in MXene research and is attributed to the diverse stacking configurations induced by intercalation.^41^

The evolution of the surface properties, i.e., chemical composition and oxidation states of Co and Mo in X–Co-Mo_2_CT_*x*_, was investigated using XPS (Figure 4, Tables S1 and S2). Upon Co impregnation, in 1-Co_Nit_-Mo_2_CT_*x*_, and in comparison with the Mo_2_CT_*x*_ profile, a decrease in Mo^4+^ species is observed (50.0–32.7 at. %), suggesting that these species may be involved in the anchoring of Co to the MXene surface.^45,46^ Similarly, in 5-Co_Nit_-Mo_2_CT_*x*,_ the concentration of Mo^4+^ diminishes completely after the introduction of 5 wt % of Co from cobalt nitrate. While there was no significant change in the fraction of Mo^6+^ between Mo_2_CT_*x*_ and in 1-Co_Nit_-Mo_2_CT_*x*_, an important increase in Mo^6+^ from ∼30 to 82.6% was observed in 5-Co_Nit_-Mo_2_CT_*x*_, suggesting that the introduction of 5 wt % of Co (nitrate precursor) in 5-Co_Nit_-Mo_2_CT_*x*_, favored the oxidation of Mo resulting in an increase in the concentration of Mo^6+^. The identity of Co was also probed by XPS (Figure 4e). In the high-resolution Co 2p spectra, the position of the major spectral line was found at 780.90 eV, signifying Co^2+^ in both 1-Co_Nit_-Mo_2_CT_*x*_ and 5-Co_Nit_-Mo_2_CT_*x*_ *.* Co/Mo atomic ratios of 0.025 and 0.285 are measured for 1-Co_Nit_-Mo_2_CT_*x*_ and 5-Co_Nit_-Mo_2_CT_*x*_, respectively.

Values indicate an enrichment of Co atoms on the material surface, more marked for the 5 wt % loading in accordance with the observed cobalt oxides enveloping MXene phase by TEM (Figure 3).

The effect of cobalt impregnation on the concentration of Mo^5+^ species, carbon defects, and the presence of O^n‑^ in the cobalt-decorated MXenes was further studied using EPR (as shown in Figure 4b,c). 1-Co_Nit_-Mo_2_CT_*x*_ was found to exhibit a much more intense signal in comparison with Mo_2_CT_*x*_, at a *g* factor of ∼2.0002.^44^ In the case of 5-Co_Nit_-Mo_2_CT_*x*_, a similar EPR profile to Mo_2_CT_*x*_ was observed, with three signals attributed to Mo^5+^_,_ oxo species (O^n–^),^47^ and carbon defects.^44^ However, the signals were weaker compared to Mo_2_CT_*x*_, with the concentration of Mo^5+^ species being less than in the parent MXene.

In summary, it has been demonstrated that upon incorporation of Co species, the MXene multilayers retained their initial morphology, with well dispersed Co^2+^ species within the MXene layers, as seen in EDS and TEM.

### Catalytic Activity in Ammonia Synthesis

The performance of MXene-based catalysts, namely, Mo_2_CT_*x*_, 1-Co_Nit_-Mo_2_CT_*x*_, and 5-Co_Nit_-Mo_2_CT_*x*_, was evaluated for ammonia synthesis at ambient pressure. The reaction was conducted under the following conditions: a flow rate of 60 mL min^–1^ of 75 vol % H_2_:N_2_ and a WHSV of 9000 mL g^–1^ h^–1^, with temperatures ranging from 250 to 400 °C without pretreatment. To confirm catalyst stability over time, the reaction was carried out using all active catalysts for at least 15 days. The results are presented in Figures 5, S3, S6 and Table 1.

**Table 1:** Summary of the Catalytic Activity of Mo_2_CT_*x*_, 1-Co_Nit_-Mo_2_CT_*x*_, 1-Co_Chl_-Mo_2_CT_*x*_, and 5-Co_Nit_-Mo_2_CT_*x*_ for Ammonia Synthesis^a^

	ammonia synthesis reaction								*E*_a_
	rate (μmol g_cata._^–1^ h^–1^)				rate (μmol g_Co_^–1^ h^–1^)				kJ/mol
	400 °C	350 °C	300 °C	250 °C	400 °C	350 °C	300 °C	250 °C	
Mo_2_CT_*x*_	23.2^b^	ND	ND	ND		ND	ND	ND	
5-Co_Nit‑_Mo_2_Ga_2_C	9.7^b^	ND	ND	ND	194	ND	ND	ND	
1-Co_Chl_-Mo_2_CT_*x*_	11	ND	ND	ND	1102	ND	ND	ND	
1-Co_Nit_-Mo_2_CT_*x*_	95	33	10	1	9499	3290	954	60	74
5-Co_Nit_-Mo_2_CT_*x*_	219	71	30	6	4380	1417	595	116	68

a Ammonia synthesis reaction under 60 mL min^–1^ of 75% H_2_ in N_2_ (BOC, 99.98%) at 400 °C and atmospheric pressure.

b Average rate over 2500 min.

**Figure 5:**
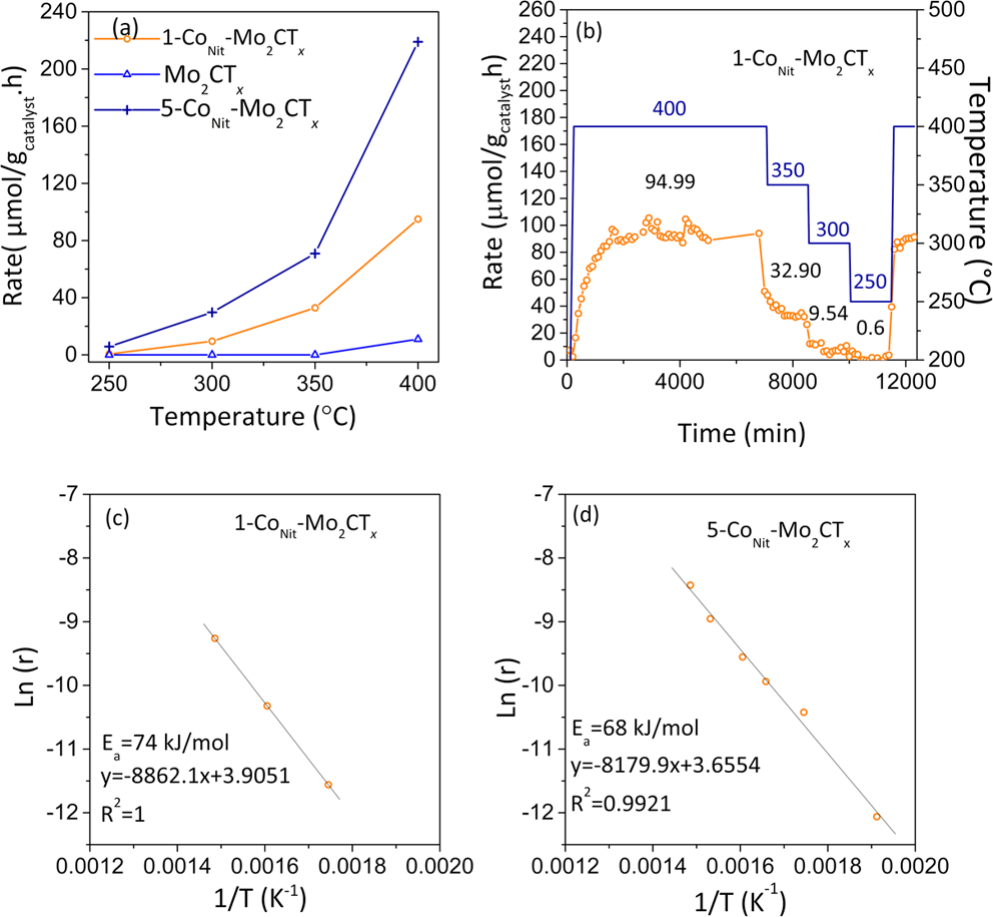
Catalytic activity in ammonia synthesis. (a) Temperature dependence of Mo_2_CT_*x*_, 1-Co_Nit_-Mo_2_CT_*x*_, and 5-Co_Nit_-Mo_2_CT_*x*_ catalytic activity for ammonia synthesis. (b) Typical ammonia yield obtained of 1-Co_Nit_-Mo_2_CT_*x*_ catalyst, (c) Arrhenius plot obtained of 1-Co_Nit_-Mo_2_CT_*x*_ catalyst, and (d) Arrhenius plot obtained of 5-Co_Nit_-Mo_2_CT_*x*_ catalyst. The reaction was performed under 60 mL min^–1^ flow rate of 75 vol % H_2_/N_2_ at 400 °C and ambient pressure.

The catalytic activity of the nondoped multilayer Mo_2_CT_*x*_ was studied at 400 °C (Figures 5a and S6, Table 1). The undecorated MXene phase demonstrated limited catalytic activity over time. Initially, the production of ammonia began at a slow rate and increased gradually, eventually reaching a maximum rate of approximately 33 μmol g_cata_.^–1^ h^–1^. However, it was observed that the material lacked stability, and the catalytic activity decreased gradually over time. After being on stream for 100 h at 400 °C, the catalytic activity was found to have decreased to ∼5 μmol g_cata_.^–1^ h^–1^ which might be related to the changes in surface composition over time (Table S1, Figure S6). Furthermore, hydrogen temperature-programmed reduction (H_2_-TPR) results of Mo_2_CT_*x*_ in 5% H_2_/Ar (50 mL min^–1^, ramp 5 °C min^–1^) indicate that the reduction process can start at temperatures as low as 300 °C (Figure S5). This was attributed in the literature to the partial defunctionalization of T_*x*_ groups.^31,48^ Upon cobalt doping, and under the same catalytic conditions, 1-Co_Nit_-Mo_2_CT_*x*_, displayed a higher catalytic activity of ∼95 μmol g_cata._^–1^ h^–1^ with respect to Mo_2_CT_*x*_. However, the steady-state rate was only attained after 25 h of reaction. Notably, once at steady-state conditions, no sign of deactivation was observed after 200 h. Remarkably, 1-Co_Nit_-Mo_2_CT_*x*_ displayed catalytic activity at temperatures below 400 °C. The ammonia synthesis rate at 350 and 300 °C was calculated to be ∼33, and ∼10 μmol g_cata._^–1^ h^–1^, respectively. While decreasing the reaction temperature resulted in a decline in the catalytic activity, the initial performance was restored upon increasing the temperature to 400 °C, with no induction time observed (Figure 5b). The use of cobalt chloride as a precursor for the preparation 1-Co_Chl_-Mo_2_CT_*x*_ led to poor catalytic activity (∼11 μmol g_cata._^–1^ h^–1^ at 400 °C, (WHSV 9000 mL g^–1^ h^–1^). The loss of catalytic activity might be related to chlorine poisoning.

Increasing the Co loading to 5 wt % resulted in further increase in the catalytic activity (5-Co_Nit_-Mo_2_CT_*x*_: ∼219 μmol g_cata._^–1^ h^–1^ at 400 °C (WHSV 9000 mL g^–1^ h^–1^). In a similar manner to 1-Co_Nit_-Mo_2_CT_*x*_, a steady state was attained after an induction time. Despite its low Co loading, 5-Co_Nit_-Mo_2_CT_*x*_ exhibits similar catalytic activity to Co_3_Mo_3_N (248 μmol g_cata._^–1^ h^–1^, WHSV 24000 mL g^–1^ h^–1^).^49^ However, unlike Co_3_Mo_3_N, no ammonolysis step or preactivation step is needed for 5-Co_Nit_-Mo_2_CT_*x*_. Moreover, the increase in the cobalt load ameliorated the performance of 5-Co_Nit_-Mo_2_CT_*x*_ allowing it to remain active at temperatures as low as 250 °C. The ammonia synthesis rate at temperatures of 250, 300, and 350 °C was calculated to be approximately 6, 30, and 71 μmol g_cata._^–1^ h^–1^, respectively. 5-Co_Nit_-Mo_2_CT_*x*_ showed no sign of deactivation even after 360 h under the stream. It is evident that MXenes improve the stability and performance of Co in ammonia synthesis at low temperatures, as Co on its own does not display catalytic activity. This becomes more apparent when the ammonia synthesis rate is normalized to the active phase loading (wt % of Co). For instance, at steady state, rates of 9499, 3290, and 945 μmol g^–1^_active phase_ h^–1^ were obtained using 1-Co_Nit_-Mo_2_CT_*x*_ at 400, 350, and 300 °C, respectively. A gradual decrease is observed when increasing the cobalt loading to 5 wt %. The ammonia synthesis rate decreased to 4380, 1417, and 595 μmolg^–1^_active phase_ h^–1^ at 400, 350, and 300 °C. However, 5-Co_Nit_-Mo_2_CT_*x*_ displayed ameliorated catalytic activity at ∼250 °C compared to 1-Co_Nit_-Mo_2_CT_*x*_. It was also observed that excess cobalt did not result in a linear increase in catalytic activity and, instead, had a negative impact on the normalized rate per gram of active phase. From these results, it can be inferred that not all the introduced cobalt is active, and only a portion of the cobalt is in direct interaction with MXene layers, contributing to the catalytic activity in ammonia synthesis.

Considering the presence of unetched Mo_2_Ga_2_C in the catalysts, an additional experiment was conducted to evaluate its potential contribution to the observed catalytic activity (Figure S7). The catalytic activity of 5-Co_Nit_-Mo_2_Ga_2_C under similar conditions to those used for the Co-impregnated Mo_2_CT_*x*_ catalysts was investigated. However, the results revealed minimal catalytic activity for Co–Mo_2_Ga_2_C, which confirms that catalytic activity observed in our study is primarily attributed to the Co–Mo_2_CT_*x*_ phase rather than the presence of unetched Mo_2_Ga_2_C.

### Co-Decorated MXene and Structure Evolution during Ammonia Synthesis

The role of cobalt in inducing ammonia catalytic activity was studied by comparing the performance of 1-Co_Nit_-Mo_2_CT_*x*_ and 5-Co_Nit_-Mo_2_CT_*x*_ against Mo_2_CT_*x*_. It is evident that without Co, Mo_2_CT_*x*_ displayed poor catalytic activity and stability in the stream, which falls in the range of activity of α-Mo_2_C under comparable conditions of T (∼47 μmol g_cata_^–1^ h^–1^).^50^ However, the catalytic activity of Co-decorated MXenes displayed an induction time where activity gradually increased before reaching a steady state. This induction time suggests that the material undergoes structural and/or surface changes, resulting in the formation of the active phase. This aspect was further investigated to gain a better understanding of the active phase. Figure 6 displays selected microscopic observations of postreaction catalysts, namely, 1-Co_Nit_-Mo_2_CT_*x*_-PR and 5-Co_Nit_-Mo_2_CT_*x*_-PR using SEM and HAADF-STEM. These catalysts were subjected to high reaction temperatures of up to 400 °C for extended periods (Figures 5b and S3). Interestingly, all catalysts, including pure Mo_2_CT_*x*_, exhibited stable multilayers that retained their accordion-like structure despite the presence of some fractures (Figure 6a,c). Furthermore, STEM-EDS showed that both catalysts maintained a high dispersion of Co species within Mo_2_CT_*x*_ (Figure 6b,d). Upon reaction, the morphology is similar for all materials tested. Therefore, there is compelling evidence suggesting that the differences in catalytic performance cannot be attributed to differences in morphology but instead are due to variations in the surface composition during the reaction. In fact, STEM-EDS analysis revealed that the surface is now populated with N adatoms upon reaction (Figure 6) in the case of 1-Co_Nit_-Mo_2_CT_*x*_–PR and 5-Co_Nit_-Mo_2_CT_*x*_–PR. The inclusion of nitrogen in the postreaction sample, Table S4, was further confirmed by elemental analysis (0.48 wt % in 1-Co_Nit_ -Mo_2_CT_*x*_–PR and 0.84 wt % in 5-Co_Nit_-Mo_2_CT_*x*_–PR). Interestingly, no significant amount of N was detected in 1-Co_Chl_-Mo_2_CT_*x*_–PR or Mo_2_CT_*x*_-PR, which are both inactive in ammonia synthesis.

**Figure 6:**
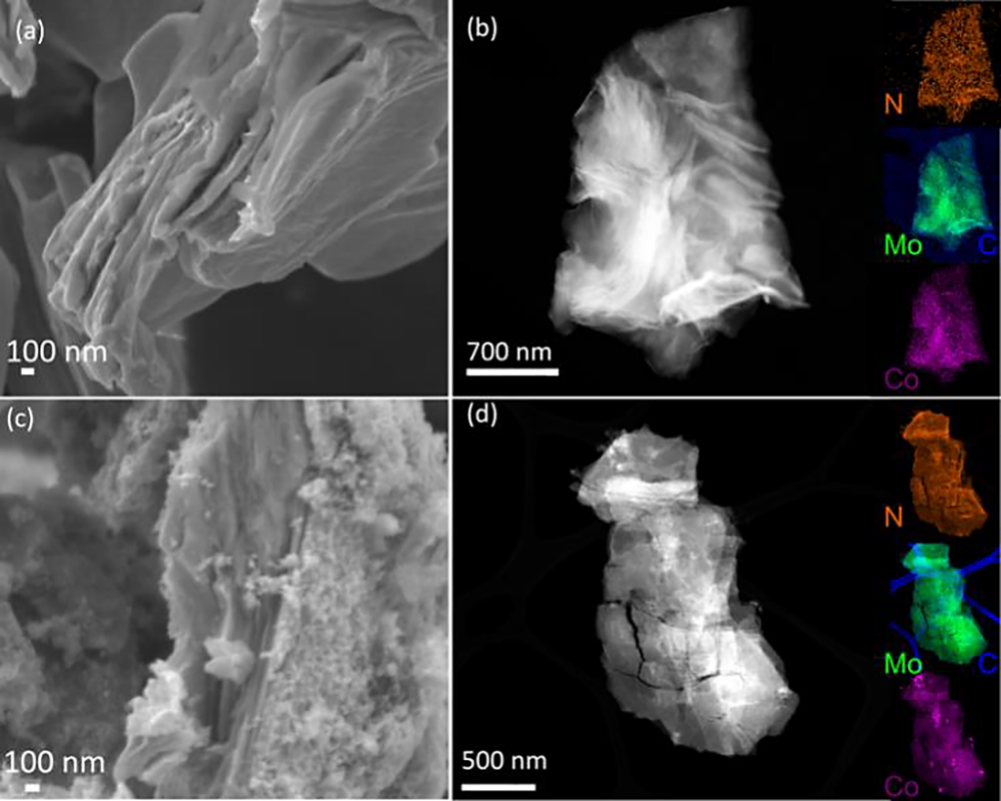
Microscopy characterizations of 1-Co_Nit_-Mo_2_CT_*x*_-PR and 5-Co_Nit_-Mo_2_CT_*x*_-PR postreaction catalysts. (a) SEM image of 1-Co_Nit_-Mo_2_CT_*x*_-PR, (b) characteristic HAADF images and the corresponding STEM-EDS of 5-Co_Nit_-Mo_2_CT_*x*_-PR. (c) SEM image of 5-Co_Nit_-Mo_2_CT_*x*_-PR and (d) characteristic HAADF images and the corresponding STEM-EDS of 1-Co_Nit_-Mo_2_CT_*x*_-PR.

To gain insight on the evolution of the surface chemical composition during the reaction, XPS and EPR analyses were performed for post reaction catalysts (Figures 7 and S4, Table S1 and S2). Upon reaction, the Mo 3d XPS spectrum features strong variations in the concentration of Mo oxidation states when compared to the as-prepared materials (Figure S4). In Mo_2_CT_*x*_, most of the Mo^6+^ and Mo^5+^ and Mo^4+^ species were reduced upon reaction to Mo^2+^, in turn becoming the predominant surface species (55.5 at. %), which is consistent with the partial defunctionalization of Mo_2_CT_*x*_ under reducing conditions, whereas, in cobalt-decorated MXenes, the redistribution of Mo species in 1-Co_Nit_-Mo_2_CT_*x*_-PR and 5-Co_Nit_-Mo_2_CT_*x*_-PR was found to be different than in Mo_2_CT_*x*_-PR (Table S1 and Figure S4). Remarkably, the addition of Co stabilizes a fraction of C–Mo-T_*x*_ species in 1-Co_Nit_-Mo_2_CT_*x*_-PR and 5-Co_Nit_-Mo_2_CT_*x*_-PR with the concentration of Mo^2+^ fraction being noticeably lower with respect to Mo_2_CT_*x*_-PR (Mo_2_CT_*x*_-PR: 55.5 at. %, 1-Co_Nit_-Mo_2_CT_*x*_-PR: 23.2 at. %, and 5-Co_Nit_-Mo_2_CT_*x*_-PR: 11.6 at. %). Further insights were obtained from the high-resolution Mo 3p spectra, which also allowed the observation of N 1s (Figure 7a). The deconvolution of 1-Co_Nit_-Mo_2_CT_*x*_-PR and 5-Co_Nit_-Mo_2_CT_*x*_-PR high-resolution Mo 3p spectra shows the presence of an additional peak at ∼397.6 ± 0.1 eV, which corresponds to the N 1s peak. The peak position is comparable to N 1s observed in molybdenum nitride phases such as in Co_3_Mo_3_N (397.9 eV) and in Mo_2_N and MoN bulk (397.8 eV)^51,52^ while NH_*x*_ species possess BE at values close to ∼400 eV.^53^ This would be consistent with partial replacement of carbon with nitrogen in the MXene phase under ammonia synthesis conditions, as observed previously.^54^ The nature of cobalt in the postreaction catalysts was also probed using XPS. Upon reaction, a radical change in Co 2p XPS profiles was observed. The spectral decomposition of Co 2p profiles showed a partial reduction of Co^2+^ into a metallic Co^0^ state. An additional peak related to the presence of a second phase was observed at 779.0 ± 0.1 and at 778.2 ± 0.1 eV in 1-Co_Nit_-Mo_2_CT_*x*_-PR and 5-Co_Nit_-Mo_2_CT_*x*_-PR, respectively. However, metallic cobalt is usually reported at 778.1 eV, while the observed BE in 1-Co_Nit_-Mo_2_CT_*x*_-PR is slightly higher, which might be due to interactions with the MXene surface. It might also imply that the extra-cobalt present in the amorphous layer enveloping the 5-Co_Nit_-Mo_2_CT_*x*_-PR phase is more prone to reduction under ammonia synthesis conditions.

**Figure 7:**
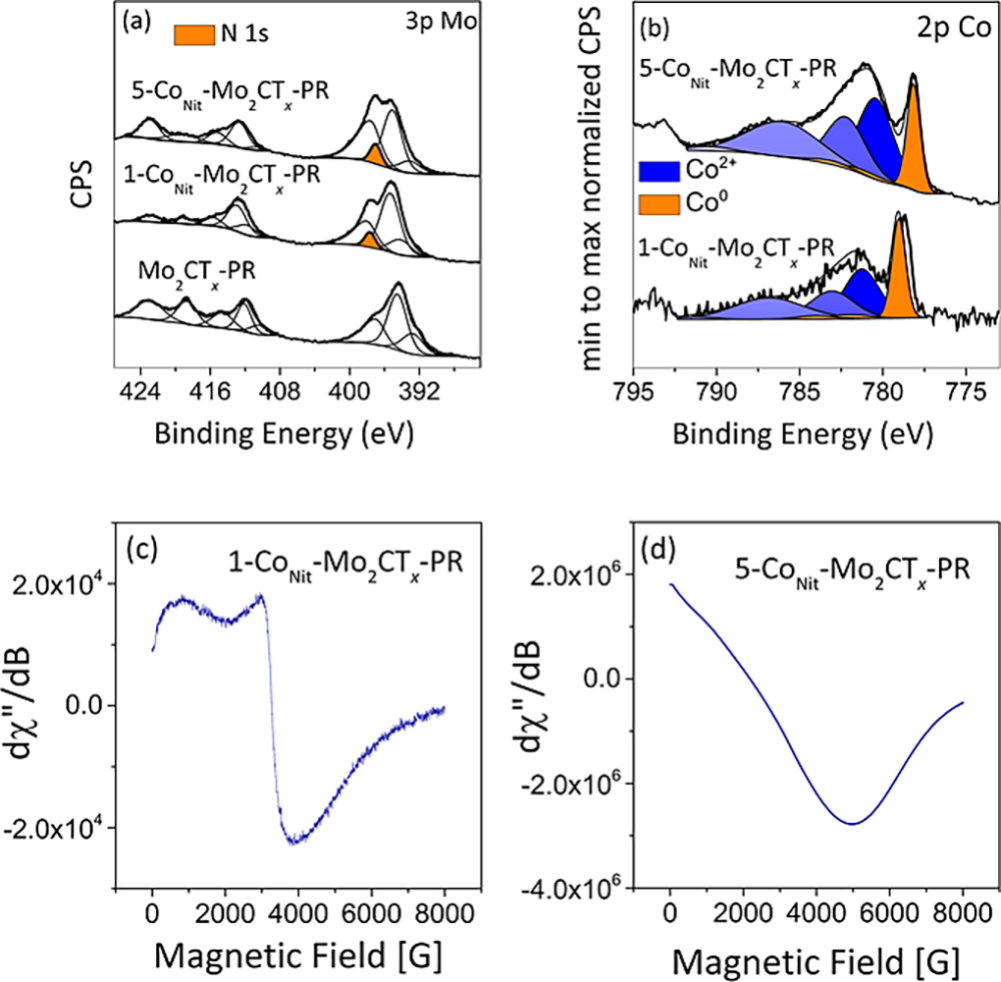
Spectroscopic characterization of postreaction Mo_2_CT_*x*_-PR, 1-Co_Nit_-Mo_2_CT_*x*_-PR, and 5-Co_Nit_-Mo_2_CT_*x*_-PR catalysts. (a) XPS spectra in the Mo 3d region of postreaction catalysts, (b) XPS spectra in the Co 2p region, (c) EPR spectrum of 1-Co_Nit_-Mo_2_CT_*x*_-PR, and (d) EPR spectrum of 5-Co_Nit_-Mo_2_CT_*x*_-PR catalysts.

For more information regarding cobalt, EPR spectra of postreaction catalysts were recorded on a wider magnetic field range (Figure 7c,d). As expected, under highly reducing conditions, signals related to Mo^5+^ and O^n–^ species, present initially in Mo_2_CT_*x*_, disappeared, whereas a signal with a *g* factor of 2.0024 attributed to carbon defects has been detected in Mo_2_CT_*x*_. The EPR spectrum of 5-Co_Nit_-Mo_2_CT_*x*_-PR (Figure 7d) was characterized by a broad line indicating ferromagnetic behavior. The magnetic property of the catalyst, upon reaction, reveals the presence of a Co^2+/^Co^0^ redox couple. The EPR spectra of the 1-Co_Nit_-Mo_2_CT_*x*_-PR (Figure 7c) were characterized by a broad line with a *g* factor of 2.15, which can be attributed to low spin Co^2+^.

## Discussion

In the current investigation, the catalytic activity of decorated Mo_2_CT_*x*_ MXene-based material was investigated for ammonia synthesis. Strong disparities in the ammonia synthesis activity at ambient pressure were observed between the nonmodified MXene (Mo_2_CT_*x*_) and Co-decorated 1-Co_Nit_-Mo_2_CT_*x*_ and 5-Co_Nit_-Mo_2_CT_*x*_. In the absence of cobalt, MXene displayed poor catalytic activity and poor stability in ammonia synthesis conditions. As demonstrated in the catalytic activity testing, the role of cobalt in inducing catalytic activity is evident where high catalytic activity at temperatures ranging between 250 and 400 °C was observed (Figure 5 and Table 1) for Co-decorated MXene. At steady state, rates of 9499, 3290, and 945 μmol g^–1^_active phase_ h^–1^ were obtained using 1-Co_Nit_-Mo_2_CT_*x*_ at 400, 350, and 300 °C, respectively, while activity at temperatures as low as 250 °C was observed at higher cobalt loading. It is worth noting that if thermodynamic equilibrium was attained, the limiting mass-normalized rate would be approximately 1768 μmol g_cata_^–1^ h^–1^, under conditions similar to those used in this study. Therefore, it appears that at 400 °C, 5-Co_Nit_-Mo_2_CT_*x*_, which displays the highest catalytic activity (219 μmol g_cata_^–1^ h^–1^), is operating at approximately ∼12% of the equilibrium value. Cobalt on its own does not exhibit significant catalytic activity or good stability in ammonia synthesis due to the low adsorption energy of N_2_ on cobalt.^5,55^ Only a limited number of supports (CeO_2_, BaTiO_3‑x_H_*x*_, BaCeO_3–x_N_*y*_H_*z*_, and BaCa­(NH_2_)_2_), modulating the electronic state of Co, promote ammonia synthesis.^17–19^ Electride-based supports characterized by strong electron donation properties and low work function (2.4 eV), enhanced the catalytic activity and stability of Co.^56^ Co on 12CaO·7Al_2_O_3_ displayed a low apparent activation energy ∼50 kJ mol^–1^ and high catalytic activity (1764 μmol g^–1^ h^–1^, 400 °C, 0.1 MPa, pressure, WHSV 18,000 mL g^–1^ h^–1^). Interestingly, Tsuji et al. reported high catalytic activity when CoMo was supported in CeO_2_.^57^ The study observed the maximum ammonia synthesis rate recorded at 400 °C, with a Co:Mo ratio of 4:6, resulting in a rate of 1.08 mmol g^–1^ h^–1^ (0.1 MPa, WHSV 72,000 mL g^–1^ h^–1^). The high catalytic activity of this system was attributed by the author to the formation of Co_3_Mo_3_N nanoparticles on the CeO_2_ support. Then, when supported on MXene, Co displayed interesting catalytic activity at 400 °C, and more importantly at temperatures as low as 300 °C. For the reaction to proceed catalytically, the strong triple bond N≡N needs to be activated via either dissociative or associative reaction mechanisms. This is clear evidence of the promoting effect of Co-MXenes on the ability to activate nitrogen at low temperatures to produce NH_3_. In our case, the apparent activation energy (*E*_a_) was found to be ∼74 and ∼68 kJ mol^–1^ for 1-Co_Nit_-Mo_2_CT_*x*_ and 5-Co_Nit_-Mo_2_CT_*x*_, respectively. These values fall within the range (40–70 kJ mol^–1^) reported for some of the most active catalysts such as Li-MT, Mn_4_N-BaH_2_, Ru/C_12_A_7_:e^–^, BaTiO_2.5_H_0.5_, which have been reported in previous studies.^58–61^ In comparison, standard Ru-based catalysts have activation energies in the range of 80–130 kJ mol^–1^. Caution should be exercised when comparing *E*_a_ of different catalysts, as reaction conditions can vary and affect the observed activation energies. However, cobalt-decorated MXene displays reasonably low activation energy.^62^ A comparison of the catalytic performance of 5-Co_Nit_-Mo_2_CT_*x*_ and selected Co-based catalysts is provided in Table S5. Analysis of the literature shows that the *E*_a_ falls in the interval of *E*_a_ obtained for active materials, and the activity positions with Co_3_Mo_3_N despite the low Co content in the material. Activity remains however lower than when cobalt is associated with hydrides or electrides, active catalysts but lacking of stability or difficult to envisage for further scaling-up.

In the current study, an induction time was observed during the catalytic activity testing and was further investigated. Interestingly, upon repeating the light-off experiment, by cooling down the active catalyst, from 400 to 200 °C and then increasing the temperature again back to 400 °C, the steady-state rate was reached much faster in the second light-off experiment (Figure S8). This can be attributed to the catalyst activation (formation of the active phase) during the induction time at the first temperature increase cycle. Subsequently, the catalytic activity demonstrates high stability, with consistent rates measured across successive runs. Structural investigation showed a similar morphology, of cobalt-decorated MXene before and after the ammonia synthesis reaction, characterized by a stable multilayer accordion-like structure (Figure 6a,c). Additionally, no change in the dispersion of Co species was observed in postreaction catalysts (Figure 6). However, when the chemical composition of MXene is investigated by elemental analysis, a partial replacement of C with N in 1-Co_Nit_-Mo_2_CT_*x*_ and 5-Co_Nit_-Mo_2_CT_*x*_ was observed and was further confirmed by the results of STEM-EDS analysis and XPS analysis. Interestingly enough, the nitridation of Mo_2_CT_*x*_ was only observed in the case of active catalysts (1-Co_Nit_-Mo_2_CT_*x*_ *-*PR and 5-Co_Nit_-Mo_2_CT_*x*_-PR), while no significant amount of N was detected in 1-Co_Chl_-Mo_2_CT_*x*_-PR or Mo_2_CT_*x*_-PR, which are both inactive in ammonia synthesis. The structural evolution of Co_Nit_-Mo_2_CT_*x*_ catalysts during ammonia synthesis shows similarities, to a certain extent, with the observed α-Mo_2_C_1–*x*_ and Co_3_Mo_3_C transformation into carbonitrides materials under ammonia synthesis conditions.^50,63^ For both catalysts, catalytic activity in ammonia synthesis was associated with the partial replacement of carbon with nitrogen atoms, a process found to occur in a topotactic and isomorphic manner. Ammonia production is further described to occur through a MvK mechanism in which lattice nitrogen is the reactive species. Thus, a critical concentration of lattice nitrogen is needed before catalytic activity reaches a steady-state regime.^63^ A possible analogy can be drawn here, in respect to α-Mo_2_C_1–*x*_ and Co_3_Mo_3_C behaviors, with potentially ammonia synthesis occurring via a MvK mechanism over the carbo-nitride derived MXene catalysts. Further studies would be needed to confirm the mechanism, considering the significant difference in the working temperature window between both families. While bulk α-Mo_2_C_1–*x*_ and Co_3_Mo_3_C becomes active only at 500 °C, Co_Nit_-Mo_2_CT_*x*_ catalysts are active at temperatures as low as 300 °C. At these lower temperatures, reaction pathway involving an associative mechanism becomes possible. In this mechanism N_2_ can be adsorbed and activated over nitrogen vacancies without surface dissociation for ammonia production.^64,65^

The correlation between the partial nitridation of 1-Co_Nit_-Mo_2_CT_*x*_ and 5-Co_Nit_-Mo_2_CT_*x*_ catalysts and their catalytic activity in ammonia synthesis infer a promoting effect of MXene on cobalt’s ability to activate N≡N at mild conditions. The activation of N≡N, which is the rate-determining step in ammonia synthesis, was reported in the literature to be facilitated by strong electron donation from the support and/or from catalytic activity promoters (LiH, BaH_2_).^21,58^ Furthermore, kinetic studies implied the possible shift of the rate-determining step from N_2_ dissociation to another elementary step in the presence of catalytic activity promoters.^21^ Considering the flexibility of MXenes’ chemical composition, the appropriate selection of transition metal and/or surface terminations (−O, −OH, F) can readily tune its surface chemistry, density of states,^32^ and work function.^33^ Taking into account that Mo_2_CT_*x*_ is just one of more than 50 MXenes reported to date, there exists an opportunity for further improving the catalytic activity in ammonia synthesis of MXene-based materials at mild conditions.

## Conclusions

In the present study, cobalt-decorated MXene was found to be a suitable heterogeneous catalyst for ammonia synthesis under mild conditions. The results demonstrate good dispersion of Co species on 2D MXene (Mo_2_CT_*x*_) multilayers and pinpoint their stability during the synthesis process. 1-Co_Nit_-Mo_2_CT_*x*_ and 5-Co_Nit_-Mo_2_CT_*x*_ catalysts displayed high catalytic activity for ammonia synthesis at ambient pressure. At steady-state conditions and 400 °C and under ambient pressure, rates of 4380 and 9500 μmol g_active phase_ h^–1^ were obtained for 5-Co_Nit_-Mo_2_CT_*x*_ and 1-Co_Nit_-Mo_2_CT_*x*_, respectively. Furthermore, both catalysts demonstrate activity in ammonia synthesis at temperatures as low as 250 °C. Remarkably, no ammonolysis or pretreatment steps were required to activate both catalysts. However, an induction time, in which the activity increased slowly, was observed. Postreaction analysis revealed partial substitution of lattice carbon with nitrogen as well as partial reduction of Co^2+^ during ammonia synthesis. However, the carbon substitution by nitrogen is limited (less than 1 wt % in 5-Co_Nit_-Mo_2_CT_*x*_). Replacement of carbon by nitrogen in the MXene lattice was only observed in the case of active materials for ammonia synthesis and not in the case of nondecorated MXene (Mo_2_CT_*x*_). The catalytic activity profiles, supported by postreaction catalysts characterization, suggest that active sites generated upon reaction might be cobalt-decorated carbonitrides Co–Mo_2_C_1−δ_N_δ_T_*x*_. The results reported in this work then demonstrate that

1. The appropriate modification of Mo_2_CT_*x*_ with Co resulted in a high-performance ammonia synthesis catalyst capable of operating under mild reaction conditions, with activity observed as low as 300 °C.
2. The catalysts are able to operate in intermittent regimes, supporting fluctuation of temperatures, and do not suffer from any deactivation after >8 days in reaction.

Considering the ease of scalability and of engineering of MXenes (that is not the case of efficient materials such as electrides imides or hydrides), it confers a great potential of these Co-MXene composites for practical ammonia synthesis.

## Materials and Methods

### Catalyst Preparation

#### Chemicals

All chemicals needed for the preparation of Mo_2_CT_*x*_ and its corresponding decorated Co–Mo_2_CT_*x*_ MXene were used with no further purification: molybdenum carbide (Mo_2_C −325 mesh, 99.5% Alfa Aesar), gallium metal (Ga, 99.9%, Alfa Aesar), cobalt nitrate hexahydrate (Co­(NO_3_)_2_·6H_2_O, 98 wt %, Sigma-Aldrich), cobalt chloride hexahydrate (CoCl_2_·6H_2_O, 98%, Sigma-Aldrich), hydrofluoric acid (HF, 50%, Sigma-Aldrich), and hydrochloric acid (HCl, 69%, Sigma-Aldrich).

#### Preparation of Precursor Phase Mo_2_Ga_2_C

Mo_2_Ga_2_C was synthesized following a modified synthesis procedure reported by Hu et al.^66^ In short, Mo_2_Ga_2_C was prepared by heating a mixture of Mo_2_C powder and Ga metal under vacuum in appropriate ratios for 15 days at 850 °C. After furnace cooling, the resulting powder was immersed in a concentrated HCl to dissolve any residual metals or intermetallics.

#### Preparation of Multilayer Mo_2_CT_*x*_

Mo_2_CT_*x*_ was synthesized by selectively etching Ga from Mo_2_Ga_2_C using 10 mL of 50 wt % of HF per 1 g of Mo_2_Ga_2_C phase at 55 °C for a duration of 4 days. Please note that HF is toxic and highly corrosive, and proper safety protocols should be used to ensure its safe handling during the synthesis of MXenes.^67^ The resulting multilayer MXene was then thoroughly washed by centrifugation until reaching neutral pH, and dried overnight under vacuum to obtain multilayer Mo_2_CT_*x*_.

#### Decoration of Multilayer Mo_2_CT_*x*_ by Co

A series of Co–Mo_2_CT_*x*_ catalysts was prepared by postsynthesis modification of Mo_2_CT_*x*_ surface by Co^2+^. In this approach, the appropriate amount of cobalt precursor (1–5 wt % load of either cobalt nitrate or cobalt chloride) was first dissolved in 1 mL of deionized water. Upon sonication, 1 g of MXene (Mo_2_CT_*x*_) was added to the mixture, and the obtained slurry was then dried in a desiccator for 4 days. The resulting material was then calcined under argon at 350 °C (heating rate 1.5 °C min^–1^) to obtain X–Co-Mo_2_CT_*x*_. Herein, the samples obtained after the calcination step are denoted as X-Co_*y*_-Mo_2_CT_*x*_, where X represents the weight percentage of Co, while y represents the cobalt precursor (Nit-nitrate; Chl-chloride). The postreaction samples are denoted as X-Co_*y*_-Mo_2_CT_*x*_-PR.

### Physical and Textural Characterizations

#### Powder XRD

Wide angle powder XRD patterns were collected using a Bruker X-ray AXS D8 Advance diffractometer in Bragg–Brentano geometry configuration fitted with a LynxEye Super Speed detector. XRD patterns were recorded with Cu Kα radiation (λ = 1.54184 Å) at 40 kV and 30 mA over a 2θ range of 5–120°, at a step size of 0.02° per step and a counting time of 7 s per step.

#### X-ray Photoelectron Spectroscopy (XPS)

XPS spectra were recorded on a Kratos Analytical AXIS Ultra DLD spectrometer employing a monochromatic Al Kα X-ray radiation (1486.6 eV), with an electron analyzer operating in a fixed pass energy of 20 eV. All BEs were referenced to the carbon signal corresponding to C–C bonding in the C 1s core level at 284.8 eV. 2p Co peaks were normalized (min to max) counts per second (CPS) for comparison purposes. To decompose our data, we followed work done by Biesinger et al.^68^ taking into account both ground state and final state effects. All experiments after the reaction were done minimizing the air contact with the sample as we previously applied for air-sensitive nitride characterization.^49^ Samples are maintained in a closed reactor until being in the glovebox where preparation is done for analysis. Transfer to the XPS analysis chamber is also done under a protected atmosphere.

#### Scanning Electron Microscopy (SEM)

SEM observations were conducted on uncoated samples using a Field Emission Gun (FEG) SEM JEOL JSM-7800F LV fitted with energy dispersive X-ray spectroscopy (EDS) system (X-Max SDD detector, Oxford Instruments). For better resolution, secondary electron images of the samples were recorded at 5 kV and low probe current. EDS analyses (maps and spectra) were performed at 5 or 15 kV accelerating voltage and 10 mm working distance to identify the elements present within the layers.

#### Transmission Electron Microscopy (TEM)

Morphology analysis was performed using a TITAN Themis 300 S/TEM equipped with a high-brightness Schottky FEG, a monochromator, and a probe aberration corrector allowing energy and spatial resolution of about 150 meV and 70 pm, respectively. The microscope is equipped with several annular dark-field detectors and a super-X detector system with four windowless silicon drift detectors for electron dispersive X-ray spectroscopy (EDS). The experiments have been performed at 300 kV with a semiconvergence angle of about 20 mrad, probe size of the order of 500 pm and probe current between 60 and 100 pA. For HAADF imaging, the *c* collection angles ranged between 50 and 200 mrad. EDS mapping was done in the spectrum imaging mode with a dwell time per pixel of about 15 μs, continuously scanning frames until the total acquisition time reached 15–20 min. All samples were deposited in powder form on a lacey carbon grid for analysis.

#### Elemental Analysis

Upon ammonia synthesis reaction, the nitrogen content in postreaction catalysts was determined using a Vario Cube elemental analyzer.

#### ICP-OES

Prior to analysis, the catalysts were dissolved in a diluted mixture of Aqua Regia and heated under microwave until complete dissolution. The concentration of Ga was determined using sequential scanning inductively coupled plasma with an optical emission spectrometer (PerkinElmer Optima 2000 DV).

#### EPR

EPR experiments were conducted at 9.863 GHz using a Bruker high-temperature resonator at ambient pressure and temperature for both pristine and postreaction catalysts. The experimental setup used a modulation amplitude of 2G and a microwave power of 5 mW. Experiments conducted after reaction are done minimizing the contact of the sample with air prior to characterization (see detail in X-ray Photoelectron Spectroscopy (XPS) section).

#### TGA

TGA was performed on Mettler Toledo TGA/DSC 3+ under 80 mL min^–1^ of N_2_ flow, heating up to 1000 °C (applying a temperature increase rate of 5 °C min^–1^).

#### Catalytic Activity

Catalytic tests were conducted in a tubular reactor operated at atmospheric pressure. In a typical reaction test, 0.4 g of catalyst was placed in the tubular quartz reactor and subjected to a 75 vol % H_2_/N_2_ (Airproducts, 99.98%) gas mixture at a total gas flow of 60 mL min^–1^. The reaction was performed at 400 °C, without any pretreatment (ammonolysis or high-temperature nitridation) under the same flow rate of 75 vol % H_2_:N_2._ Ammonia production was determined by measurement of the decrease in conductivity of a 200 mL 0.0018 M H_2_SO_4_ solution through which the reactor effluent stream flowed and was monitored as a function of reaction time. Reaction at 400 °C was conducted to reach a stationary regime, before decreasing temperature stepwise by 50 °C until no significant ammonia production was measured. For each temperature, the reaction was conducted for at least 20 h to reach a stationary regime.

## Supplementary Material



## Notes

Thermogravimetric analysis of Mo_2_CT_*x*_, XPS data prior and postreaction, catalytic test results, and elemental analysis (PDF)

## Abbreviations

H–B: Haber Bosch
WHSV: weight hourly space velocity
DFT: density functional theory
MvK: Mars-van Krevelen
XPS: X-ray photoelectron spectroscopy
XRD: X-ray diffraction
EPR: electronic paramagnetic resonance
SEM: scanning electronic microscopy
HAADF: high-angle annular dark-field imaging
FFT: fast Fourier transform
STEM-EDS: scanning transmission electron microscopy-energy dispersive X-ray spectroscopy
ND: not detected
*E*_a_: activation energy
BE: binding energy
CPS: counts per second
FEG: field emission gun
TGA: thermogravimetry analysis
Nit: nitrate
Chl: chloride
