# Supplementary material for: Unlocking the Potential of MXene in Catalysis: Decorated Mo2CT x Catalyst for Ammonia Synthesis under Mild Conditions
Source: J Am Chem Soc. 2024 Jul 12;146(29):20033–44. doi: 10.1021/jacs.4c03875 (PMC12239075; doi:10.1021/jacs.4c03875)
Supplement: Supplementary file 1 [file ja4c03875_si_001.pdf]

Supplementary information

Unlocking the Potential of MXene in Catalysis: Decorated Mo_2CT_x Catalyst for Ammonia Synthesis under Mild Conditions

Amanda Sfeir[a], Christopher E. Shuck[b], Alexandre Fadel[c], Maya Marinova[c], Hervé Vezin[d], Jean-Philippe Dacquin[a], Yury Gogotsi[b]*, Sébastien Royer[a]*, and Said Laassiri[e]*

[a] Université de Lille, CNRS, ENSCL, Centrale Lille, Univ. Artois, UMR 8181-UCCS-Unité de Catalyse et de Chimie du Solide, F-59000 Lille, France.

[b] A.J. Drexel Nanomaterials Institute and Department of Materials Science and Engineering, Drexel University, Philadelphia, PA 19104, USA

[c] Université de Lille, CNRS, INRA, Centrale Lille, Université Artois, FR 2638 – IMEC – Institut Michel-Eugène Chevreul, 59000 Lille, France

[d] Laboratoire de Spectroscopie pour Les Interactions La Réactivité et L'Environnement Université de Lille, UMR CNRS 8516-LASIRE, 59000 Lille, France

[e] Chemical & Biochemical Sciences, Green Process Engineering (CBS), Mohammed VI Polytechnic University, UM6P, 43150, Benguerir, Morocco

* Corresponding authors: gogotsi@drexel.edu, sebastien.royer@univ-lille.fr, said.laassiri@um6p.ma

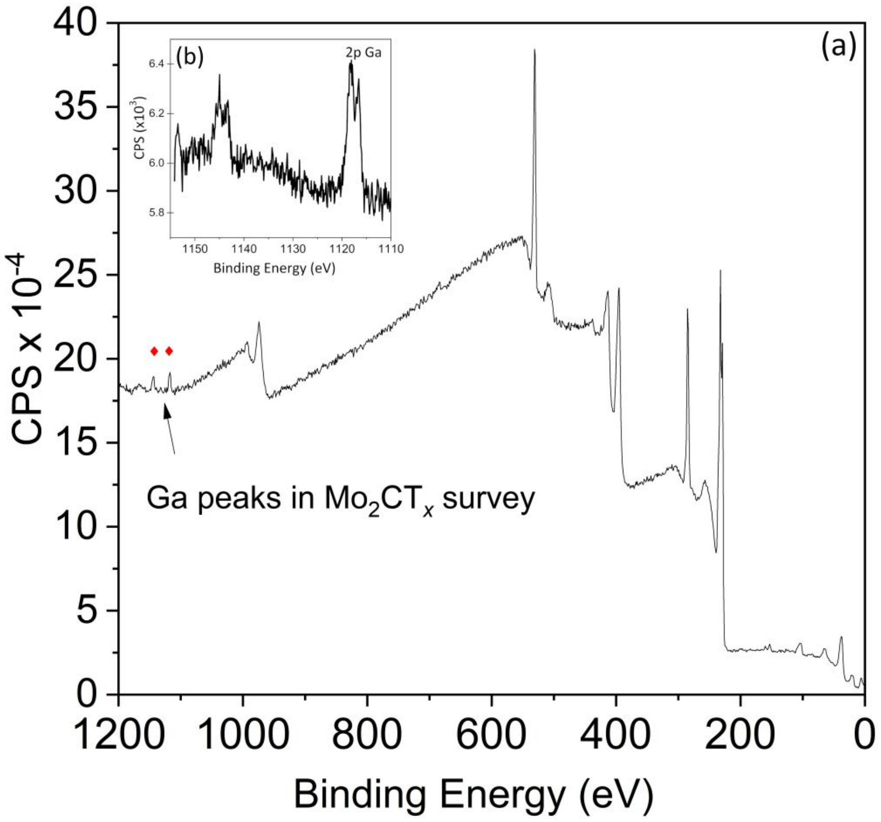

Figure S1 XPS spectra of Mo_2CT_x. (a) survey spectrum (b) high-resolution XPS spectrum of Ga 2p**.**

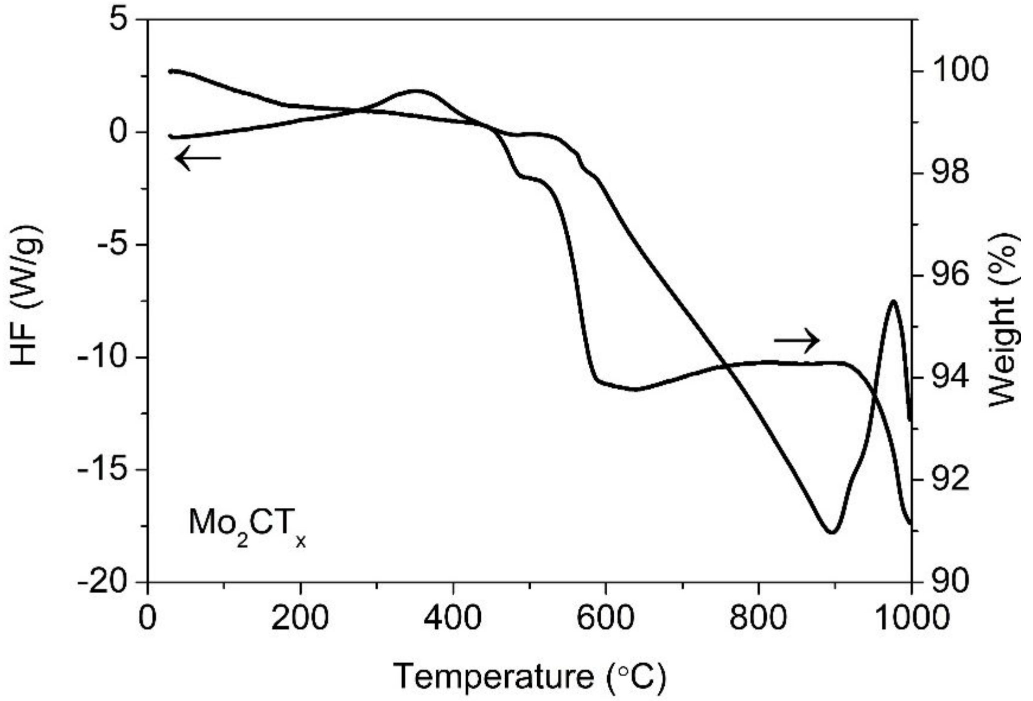

Figure S2. Thermogravimetric analysis of Mo_2CT_x.

Table S1. Position of 3d Mo peaks of catalysts before and after reaction.

	Mo Peaks								Surface Composition			
	Mo^{6+}		Mo^{5+}		Mo^{4+}		Mo^{2+}		Mo^{6+} (%)	Mo^{5+} (%)	Mo^{4+} (%)	Mo^{2+} (%)
	$3d_{3/2}$	$3d_{5/2}$	$3d_{3/2}$	$3d_{5/2}$	$3d_{3/2}$	$3d_{5/2}$	$3d_{3/2}$	$3d_{5/2}$				
Mo_2CT_x	235.82	232.62	234.02	230.82	232.65	229.45	231.60	228.4	27.5	12.6	50.0	9.9
Mo_2CT_x-PR	235.57	232.37	233.90	230.70	232.46	229.26	231.34	228.14	8.2	3.6	32.7	55.5
1-Co_{Chl} -Mo_2CT_x	235.70	232.50	234.80	230.80	232.72	229.52	231.70	228.50	19.4	19.3	50.1	11.2
1-Co_{Chl} -Mo_2CT_x -PR	235.78	232.58	234.29	231.09	232.53	229.33	231.66	228.46	10.1	6.7	69.4	13.8
1-Co_{Nit} -Mo_2CT_x	235.91	232.71	234.12	230.92	232.86	229.66	231.80	228.60	33.0	30.0	32.7	4.3
1-Co_{Nit} -Mo_2CT_x -PR	236.05	232.85	234.37	231.17	232.67	229.47	231.91	228.71	12.3	11.3	53.2	23.2
5-Co_{Nit} -Mo_2CT_x	235.77	232.57	234.00	230.80	-	-	232.20	229.00	82.6	9.1	-	8.3
5-Co_{Nit} -Mo_2CT_x -PR	235.51	232.31	234.01	230.81	232.48	229.28	231.80	228.60	24.2	10.0	54.2	11.6

Table S2. Position of 2p Co peaks of catalysts before and post reaction.

	Co peaks							Surface composition	
	**Co^{2+}**	**Co^{2+}**	**Co^{2+}**	**Co^{2+}**	**Co^0**	**Co^0**	**Co^0**	**Co^{2+}**	**Co^0**
	$2p_{3/2}$	$2p_{3/2}$	$2p_{3/2}$	$2p_{3/2}$	$2p_{3/2}$	$2p_{3/2}$	$2p_{3/2}$	(%)	(%)
1-Co_{Nit}-Mo_2CT_x	780.90	782.70	786.49	790.89	-	-	-	100	-
1-Co_{Nit}-Mo_2CT_x-PR	781.28	783.08	786.87	791.27	779.05	782.05	784.05	73.60	26.40
5-Co_{Nit}-Mo_2CT_x	780.90	782.70	786.49	790.89	-	-	-	100	-
5-Co_{Nit}-Mo_2CT_x-PR	780.42	782.22	786.01	790.41	778.16	781.16	783.16	81.05	18.95

Table S3. Position of 1s O peaks of catalysts before and post reaction.

	O Peaks				Surface Composition			
	**M-O**	**Mo_2CO_x**	**$Mo_2C(OH)_x$**	**H_2O_{ads}**	**M-O**	**Mo_2CO_x**	**$Mo_2C(OH)_x$**	**H_2O_{ads}**
	1s	1s	1s	1s	(%)	(%)	(%)	(%)
Mo_2CT_x	530.30	530.90	532.12	533.44	30.85	29.64	23.96	15.56
Mo_2CT_x-PR	530.19	531.08	532.19	533.60	17.72	35.61	14.22	32.45
1-Co_{Chl}-Mo_2CT_x	530.30	530.93	532.40	533.60	14.01	39.05	22.06	24.87
1-Co_{Chl}-Mo_2CT_x-PR	530.30	531.03	532.40	533.60	14.58	37.37	15.82	32.23
1-Co_{Nit}-Mo_2CT_x	530.30	530.97	532.40	533.60	3.06	69.02	18.37	9.55
1-Co_{Nit}-Mo_2CT_x-PR	530.30	530.94	532.00	533.45	4.89	57.21	26.98	10.95
5-Co_{Nit}-Mo_2CT_x	529.97	530.90	533.35	532.33	13.08	68.77	11.77	6.38
5-Co_{Nit}-Mo_2CT_x-PR	530.11	530.90	533.60	532.00	20.88	49.12	21.15	8.86

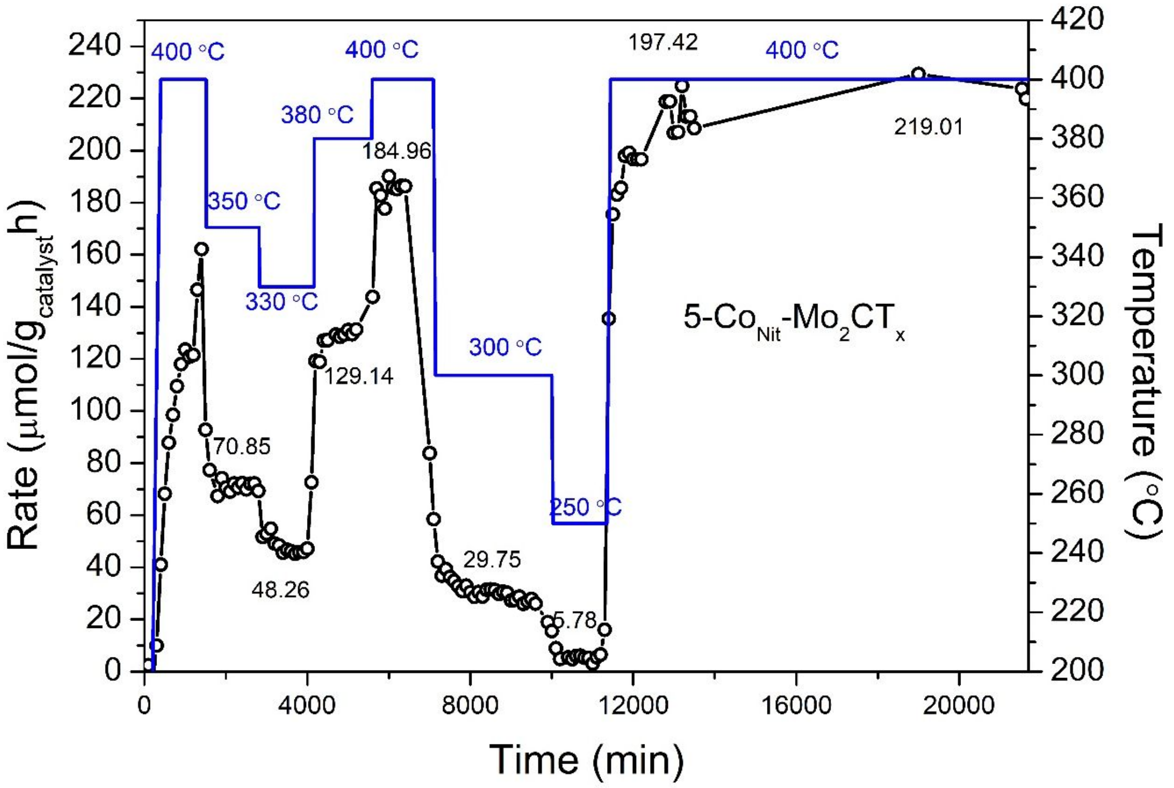

Figure S3 Typical cumulative ammonia yield obtained of 5-Co_{Nit}-Mo_2CT_x catalyst.

Table S4. Elemental Analysis (CHNS) on catalysts before and after reaction.

	C/ (wt.%)	H/ (wt.%)	N/ (wt.%)
Mo_2CT_x	3.89	0.105	0
Mo_2CT_x-PR	4.86	0.026	0.04
1-Co_{Chl}-Mo_2CT_x	5.2	0.039	0.02
1-Co_{Chl}-Mo_2CT_x-PR	5.17	0.029	0.04
1-Co_{Nit}-Mo_2CT_x	5.09	0.023	0.03
1-Co_{Nit}-Mo_2CT_x-PR	4.68	0.028	0.48
5-Co_{Nit}-Mo_2CT_x	3.72	0.03	0.06
5-Co_{Nit}-Mo_2CT_x-PR	3.43	0.036	0.84

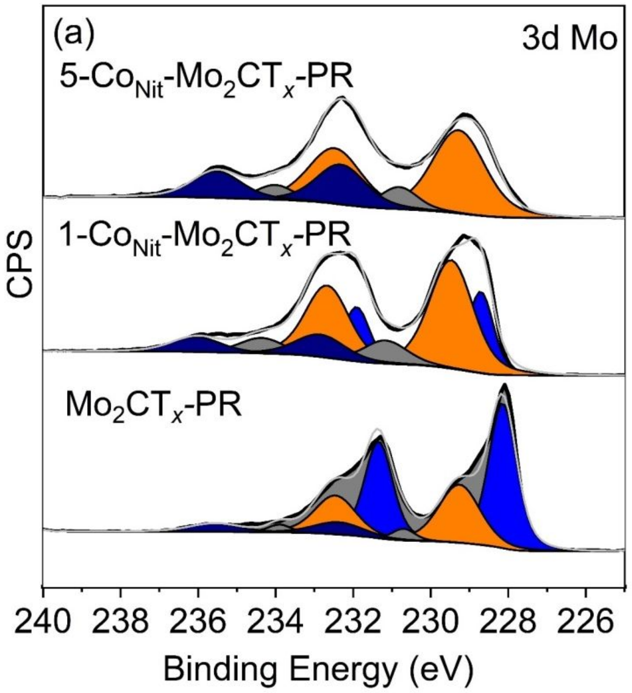

Figure S4 High resolution XPS spectra in the Mo 3d region of post-reaction catalysts.

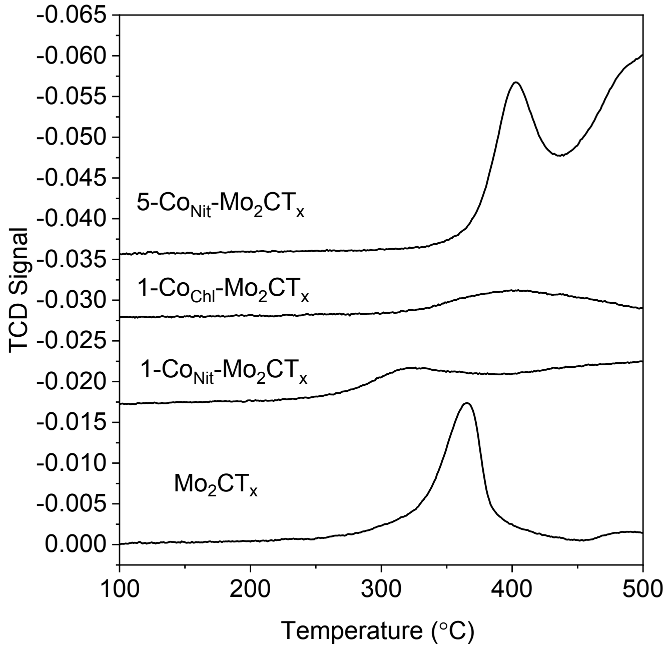

Figure S5 Temperature-programmed reduction (H_2-TPR) of Mo_2CT_x, 1-Co_{Nit}- Mo_2CT_x, 1-Co_{Chl}- Mo_2CT_x, and 5-Co_{Chl}- Mo_2CT_x in 5% H_2/Ar (50 ml.min^{-1}, ramp 5 °C.min^{-1}).

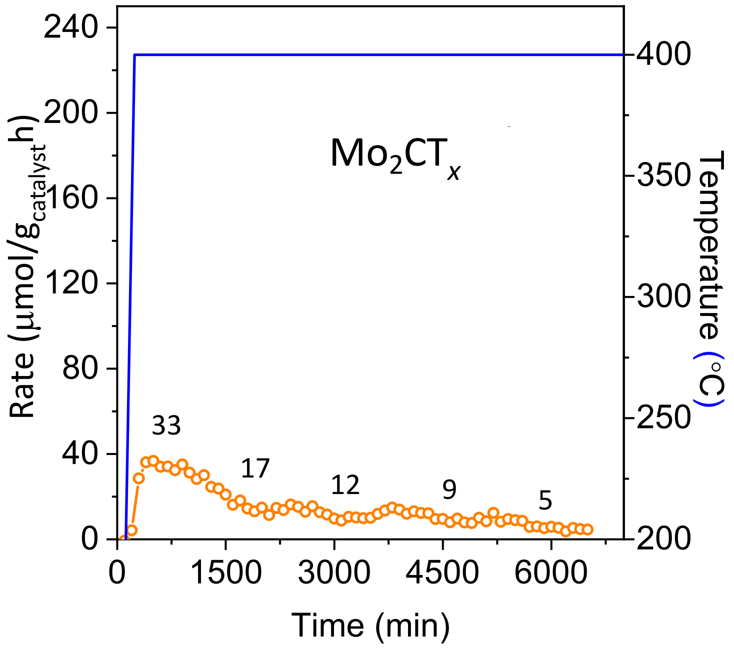

Figure S6 Ammonia yield obtained on a Mo_2CT_x catalyst. The reaction was performed under 60 mL min^{-1} flow rate of 75 vol.% H_2/N_2 at 400 °C and ambient pressure.

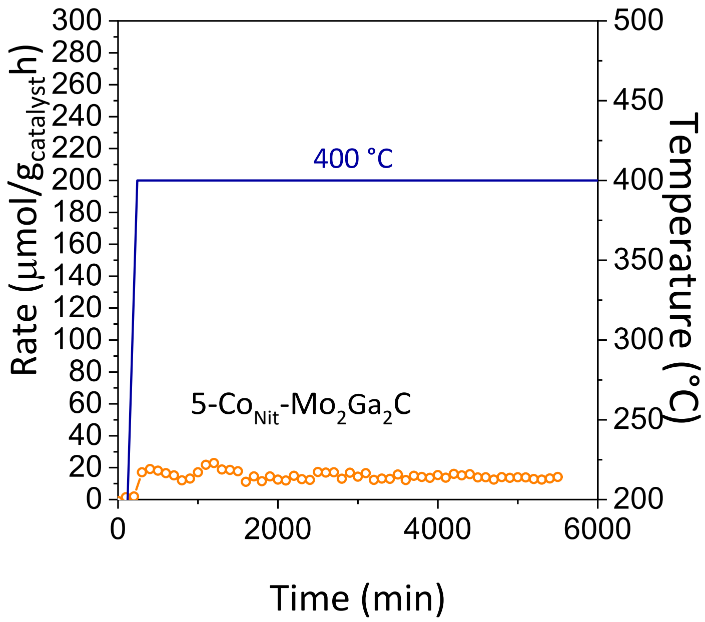

Figure S7 Ammonia yield obtained on 5-Co$_{Nit}$-Mo_2Ga_2C catalyst. The reaction was performed under 60 mL min^{-1} flow rate of 75 vol.% H_2/N_2 at 400 °C and ambient pressure.

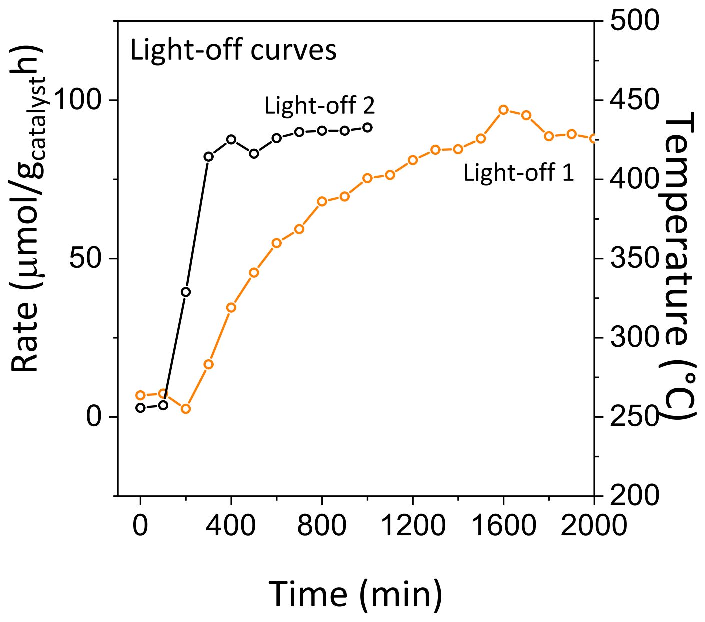

Figure S8 Light-off curve off ammonia synthesis obtained of 1-Co_{Nit}- Mo_2CT_x catalyst. The reaction is conducted under 60 mL min^{-1} flow rate of 75 vol.% H_2/N_2 at 400 °C and ambient pressure.

Table S5. Comparison of the apparent activation energies (E_a) of various Cobalt based catalysts

**Catalyst**	**Reaction conditions**		**Co/ %wt**	**Rate/ µmol. $g_{cata}^{-1}h^{-1}$**	**E_a/ kJ mol^{-1}**	**WHSV/ mL g^{-1} h^{-1}**	**Refs.**
	**Temperature/ °C**	**Pressure/ MPa**					
**5-Co_{Nit}-Mo_2CT_x**	**400**	**0.1**	**5**	**219**	**68**	**9000**	**This work**
**Co_3Mo_3N**	**400**	**0.1**		**165**	**n.d.**	**9 000**	**S1**
**Co_3Mo_3C**	**400**	**0.1**	**28.5**	**Not active**	**n.d.**	**12 000**	**S2**
**Co_3Mo_3C**	**500**	**0.1**	**28.5**	**461 (500°C)**	**n.d.**	**12 000**	**S2**
**Co-Mo/Ce**	**400**	**0.1**	**3.5**	**1.08*(400°C)**	**n.d.**	**72 000**	**S3**
**Co/SrNH**	**400**	**0.9**	**1.5**	**779.2*(400°C)**	**51.8**	**36,000**	**S4**
**Co/C12A7:e^-**	**340**	**0.1**	**2.6**	**912 (340°C)**	**49.5**	**18000**	**S5**
**Co/$BaAl_2O_{4-x}H_y$**	**340**	**0.9**	**4.7**	**8288 (340°C)**	**48.9**	**36000**	**S6**
**LaCoSi**	**400**	**0.1**	**26.1**	**1,250 (400°C)**	**42**	**36000**	**S7**
**Co-LiH**	**350**	**1.0**	**59.8**	**12000 (350°C)**	**52.1**	**60000**	**S8**

*** activity expressed in mmol·g_{Co}^{-1}·h^{-1}**
n.d. not determined

References

(S1) Hargreaves, J. S. J.; McKay, D. A comparison of the reactivity of lattice nitrogen in Co3Mo3N and Ni2Mo3N catalysts. *J. Mol. Catal. A: Chem.* **2009**, *305* (1), 125-129. DOI: https://doi.org/10.1016/j.molcata.2008.08.006.

(S2) AlShibane, I.; Daisley, A.; Hargreaves, J. S. J.; Hector, A. L.; Laassiri, S.; Rico, J. L.; Smith, R. I. The Role of Composition for Cobalt Molybdenum Carbide in Ammonia Synthesis. *ACS Sustain. Chem. Eng.* **2017**, *5* (10), 9214-9222. DOI: 10.1021/acssuschemeng.7b02168.

(S3) Tsuji, Y.; Ogasawara, K.; Kitano, M.; Kishida, K.; Abe, H.; Niwa, Y.; Yokoyama, T.; Hara, M.; Hosono, H. Control of nitrogen activation ability by Co-Mo bimetallic nanoparticle catalysts prepared via sodium naphthalenide-reduction. *J. Catal.* **2018**, *364*, 31-39. DOI: https://doi.org/10.1016/j.jcat.2018.04.029.

(S4) Li, Z.; Lu, Y.; Li, J.; Xu, M.; Qi, Y.; Park, S.-W.; Kitano, M.; Hosono, H.; Chen, J.-S.; Ye, T.-N. Multiple reaction pathway on alkaline earth imide supported catalysts for efficient ammonia synthesis. *Nat. Commun.* **2023**, *14* (1), 6373. DOI: 10.1038/s41467-023-42050-7.

(S5) Inoue, Y.; Kitano, M.; Tokunari, M.; Taniguchi, T.; Ooya, K.; Abe, H.; Niwa, Y.; Sasase, M.; Hara, M.; Hosono, H. Direct Activation of Cobalt Catalyst by 12CaO·7Al2O3 Electride for Ammonia Synthesis. *ACS Catal.* **2019**, *9* (3), 1670-1679. DOI: 10.1021/acscatal.8b03650.

(S6) Jiang, Y.; Takashima, R.; Nakao, T.; Miyazaki, M.; Lu, Y.; Sasase, M.; Niwa, Y.; Abe, H.; Kitano, M.; Hosono, H. Boosted Activity of Cobalt Catalysts for Ammonia Synthesis with BaAl2O4–xHy Electrides. *J. Am. Chem. Soc.* **2023**, *145* (19), 10669-10680. DOI: 10.1021/jacs.3c01074.

(S7) Gong, Y.; Wu, J.; Kitano, M.; Wang, J.; Ye, T.-N.; Li, J.; Kobayashi, Y.; Kishida, K.; Abe, H.; Niwa, Y.; et al. Ternary intermetallic LaCoSi as a catalyst for N2 activation. *Nat. Catal.* **2018**, *1* (3), 178-185. DOI: 10.1038/s41929-017-0022-0.

(S8) Wang, P.; Chang, F.; Gao, W.; Guo, J.; Wu, G.; He, T.; Chen, P. Breaking scaling relations to achieve low-temperature ammonia synthesis through LiH-mediated nitrogen transfer and hydrogenation. *Nat. Chem.* **2017**, *9* (1), 64-70. DOI: 10.1038/nchem.2595.
